# Correlation of microarray-based breast cancer molecular subtypes and clinical outcomes: implications for treatment optimization

**DOI:** 10.1186/1471-2407-11-143

**Published:** 2011-04-18

**Authors:** Kuo-Jang Kao, Kai-Ming Chang, Hui-Chi Hsu, Andrew T Huang

**Affiliations:** 1 Department of Research, Koo Foundation SYS Cancer Center, 125 Lih-Der Road, Taipei 112, Taiwan; 2 Department of Medicine, Duke University Medical Center, Durham, NC 27710, USA

## Abstract

**Background:**

Optimizing treatment through microarray-based molecular subtyping is a promising method to address the problem of heterogeneity in breast cancer; however, current application is restricted to prediction of distant recurrence risk. This study investigated whether breast cancer molecular subtyping according to its global intrinsic biology could be used for treatment customization.

**Methods:**

Gene expression profiling was conducted on fresh frozen breast cancer tissue collected from 327 patients in conjunction with thoroughly documented clinical data. A method of molecular subtyping based on 783 probe-sets was established and validated. Statistical analysis was performed to correlate molecular subtypes with survival outcome and adjuvant chemotherapy regimens. Heterogeneity of molecular subtypes within groups sharing the same distant recurrence risk predicted by genes of the Oncotype and MammaPrint predictors was studied.

**Results:**

We identified six molecular subtypes of breast cancer demonstrating distinctive molecular and clinical characteristics. These six subtypes showed similarities and significant differences from the Perou-Sørlie intrinsic types. Subtype I breast cancer was in concordance with chemosensitive basal-like intrinsic type. Adjuvant chemotherapy of lower intensity with CMF yielded survival outcome similar to those of CAF in this subtype. Subtype IV breast cancer was positive for ER with a full-range expression of HER2, responding poorly to CMF; however, this subtype showed excellent survival when treated with CAF. Reduced expression of a gene associated with methotrexate sensitivity in subtype IV was the likely reason for poor response to methotrexate. All subtype V breast cancer was positive for ER and had excellent long-term survival with hormonal therapy alone following surgery and/or radiation therapy. Adjuvant chemotherapy did not provide any survival benefit in early stages of subtype V patients. Subtype V was consistent with a unique subset of luminal A intrinsic type. When molecular subtypes were correlated with recurrence risk predicted by genes of Oncotype and MammaPrint predictors, a significant degree of heterogeneity within the same risk group was noted. This heterogeneity was distributed over several subtypes, suggesting that patients in the same risk groups require different treatment approaches.

**Conclusions:**

Our results indicate that the molecular subtypes established in this study can be utilized for customization of breast cancer treatment.

## Background

The advent of high-density DNA microarray technology has enabled researchers to measure the expression of a large number of genes in breast cancer and identify its molecular subtypes [1-3]. In a seminal study by Perou et al. [1], it was shown that breast cancer could be divided into four intrinsic types according to their gene expression profiles. A later study revised this to six intrinsic types [2]. Similar results were obtained when the same set of classifier genes was applied to other breast cancer datasets [4-6]. Other studies have also identified gene expression signatures applicable to the prediction of risk associated with regional recurrence, distant metastasis, and survival [6-11].

Despite these advancements related to the intrinsic types of breast cancer, the direct clinical application of molecular subtypes based on global intrinsic biology has yet to be realized. The clinical trials that have been launched recently are based on prediction of distant recurrence risk through gene expression [12,13]. These approaches do not address the likely heterogeneity of breast cancer within groups sharing the same predicted risk. Thus, the approaches based on prediction of distant recurrence risk have not taken full advantage of gene expression profiles to customize breast cancer treatment according to molecular subtypes. Studies on how microarray-based molecular subtypes could be correlated with outcomes of various specific treatment regimes are sorely needed.

In addition, the existence of a specific subset of breast cancer that can benefit most from anthracycline is still a contentious issue. It remains uncertain whether patients of this subset could be reliably identified according to the over-expression of *HER2 *and *TOP2A *genes [14-17]. The possible identification of this subset of breast cancer patients through molecular subtypes classified according to high dimensional gene expression remains unexplored.

In seeking answers to these questions, we conducted a retrospective gene expression profiling study on breast cancer tissues collected from patients who had received treatment and long-term clinical follow-up at our institution.

## Methods

### Patients and Samples

Fresh frozen breast cancer tissue from every third patient diagnosed and treated between 1991 and 2004 at the Koo Foundation Sun-Yat-Sen Cancer Center (KFSYSCC) were randomly selected for the study. Patients with follow-up periods shorter than three years were excluded, with the exception of those who died of the disease within three years of the initial treatment. In cases of ineligibility, the following sample was selected. The selected tissue samples spanned the major transition periods of adjuvant chemotherapy from CMF (cyclophosphamide, methotrexate and fluorouracil) to CAF (cyclophosphamide, doxorubicin, fluorouracil) and to taxane-based regimens. Four hundred forty seven samples were obtained, but 135 samples were excluded due to insufficient RNA (n = 1), poor RNA quality (n = 116), or unacceptable microarray quality (n = 18). A total of 312 samples were eligible for the study (Cohort 1). Gene expression profiles of an additional 15 lobular breast carcinoma samples, collected between 1999 and 2004 and previously studied, were also included (Cohort 2). All patients were treated by a multidisciplinary team according to the guidelines consistent with the National Comprehensive Cancer Network [18]. Following modified radical mastectomy or breast-conserving surgery plus dissection of axillary nodes, patients received radiotherapy, adjuvant chemotherapy, and/or hormonal therapy, if indicated. Neoadjuvant chemotherapy was administered to patients with locally advanced disease. The study was approved by the institutional review board (ID number 20020128A) and ethical approval was obtained from the same board for samples without obtainable informed consent.

### mRNA Transcript Profiling

Total RNA was isolated using Trizol (Invitrogen, Carlsbad, CA) and purified with the RNeasy Mini Kit (Qiagen, Valencia, CA). RNA quality was assessed using an RNA 6000 Nano Kit and an Agilent 2100 Bioanalyzer (Agilent Technologies, Waldbronn, Germany). The RNA samples used for the study had an average RNA Integrity Number of 7.85 ± 0.99 (mean ± SD). Hybridization targets were prepared from total RNA according to the Affymetrix protocol and hybridized to U133 plus 2.0 arrays. The expression intensity of each gene was scaled to a trimmed-mean of 500, logarithmically transformed to base 2 and normalized using quantile normalization. The dataset and MIAME compliant information had been deposited in the GEO database (GSE20685).

### Breast Cancer Molecular Subtyping

Although the classifier genes of Perou-Sørlie intrinsic types [2] could be applied to our datasets, such direct application, crossing to a different microarray platform for molecular subtyping could compromise the robustness and accuracy of the classification. To establish a reliable classification method specific to the Affymterix microarray platform, we decided to develop and validate a platform-specific methodology for the molecular subtyping of breast cancer. From the literature, we selected 23 pivotal genes known to play important roles in the biology of breast cancer (Additional file 1, Table S1), and subsequently conducted linear and quadratic correlations with each of the 23 pivotal genes for all probe-sets. The probe-sets showing significant degree of correlation with any of the pivotal genes were further selected according to their expression intensities, range of expression, and density plot kurtosis. Finally, 783 probe-sets were selected and used for molecular subtyping (Additional file 1, Table S2). The procedures associated with probe-set selection and two-step *k *means clustering for classification are detailed in the methodology in the supplemental files (Additional File 2).

### Validation of Breast Cancer Molecular Subtypes

The genes used for our molecular subtyping were applied to three independent datasets for validation [10,19,20]. Genes corresponding to our classification probe-sets were identified in the published datasets. If one probe-set was mapped to multiple genes in the independent datasets, the average intensity was calculated and applied. Centroid analysis was used to determine subtypes of breast cancer [5]. Hierarchical clustering analysis was conducted to examine whether the same subtypes identified in ours and three other independent datasets shared the same differential expression patterns for genes of wound-response [9], tumor stromal reaction [21], tumor vascular endothelial normalization [22,23], and cell cycle proliferation (Additional file 1, Table S3).

### Correlation Studies

In addition to examining the relationship between the molecular subtypes of breast cancer identified in this study and various clinical parameters, our classifier genes were also applied to the other two published independent breast cancer datasets for confirmation [10,24]. In addition, we used the reported genes of the OncotypeDX [8] and MammaPrint [3] predictors to assess the risk of distant recurrence for cases in all three datasets. For prediction of recurrence risk by the genes of OncotypeDx predictor, we adopted the same statistical predictive model used by Paik et al. [8]. The molecular subtypes were correlated with the predicted risk of recurrence. The procedures of these studies are detailed in the methodology section of Additional file 2.

### Determination of *ER*, *PR *and *HER2 *Statuses by Microarray

To quantitatively determine the status of *ER*, *PR*, and *HER2*, we used the intensity of gene expression measured by a microarray, because not all of the patients had results for *ER*, *PR*, and *HER2 *by immunohistochemistry (IHC). The values of gene expression used to determine the positive or negative status of *ER, PR*, and *HER2 *were based on density plots of 312 breast cancer samples in Cohort 1 (Additional file 3, Figure S1). Bimodal distribution was observed for all three genes, and the cut-points were statistically determined, according to the method described in the methodology section of Additional file 2. Studies into the correlation between the results of IHC and gene expression for the status of *ER *and *HER2 *showed significant positive correlations (Additional file 3, Figure S2). This finding supports the approach of using the intensity of gene expression to determine the status of *ER*, *PR*, and *HER2*.

### Statistical Methods

All statistical analysis was conducted using the SAS/STAT software (ver. 9.1.3) (SAS Institute, Inc.) and the R software package (v2.6) from Bioconductor (http://www.bioconductor.org). Heat-maps were generated using the R software (v2.9.1). All comparisons of survival were performed using the log-rank test and all Kaplan-Meier survival curves were plotted using S-Plus software (ver. 6.0.2).

## Results

### Clinical Characteristics of Breast Cancer Patients

Table 1 summarizes the clinical characteristics of the 327 patients in our cohorts. Fifteen samples in cohort 2 were lobular carcinomas. Consequently, most breast cancer samples in cohort 2 were positive for *ER *and *PR *with a lower nuclear grade and fewer *HER2 *positive cases.

**Table 1:** Clinical characteristics of patients included in the study.

	Cohort 1 (n = 312)		Cohort 2 (n = 15)	
	No.	%	No.	15
**Age at diagnosis**				
< 50 yr	197	63%	6	40%
≥ 50 yr	115	37%	9	60%
**Treatment year**				
Before 1997	125	40%	0	0%
After 1997	187	60%	15	100%
**TNM Stage**				
I	67	21%	2	13%
II	139	45%	8	53%
III	98	31%	5	33%
IV	8	3%	0	0%
**Positive Lymph Nodes**				
0	132	42%	5	33%
1-3	83	27%	5	33%
4-9	58	19%	3	20%
≥ 10	35	11%	2	13%
Unavailable	4	1%		
**Nuclear Grade**				
1	25	8%	8	53%
2	83	27%	7	47%
3	202	65%	0	0%
Unavailable	2	1%		
ER *				
ER+	190	61%	14	93%
ER-	122	39%	1	7%
HER2*				
HER2+	74	24%	1	7%
HER2-	238	76%	14	93%
PR*				
PR+	244	78%	14	93%
PR-	68	22%	1	7%
**Treatment**				
Neoadjuvant Chemotherapy	31	10%	0	0%
Adjuvant Chemotherapy	220	71%	12	80%
Radiation Therapy	133	43%	8	53%
Hormonal Rx	210	67%	14	93%
No chemotherapy	50	16%	3	20%

*: ER, HER2 and PR status were determined according to microarray data.

### Clinical Characteristics of Molecular Subtypes of Breast cancer

**Figure 1:**
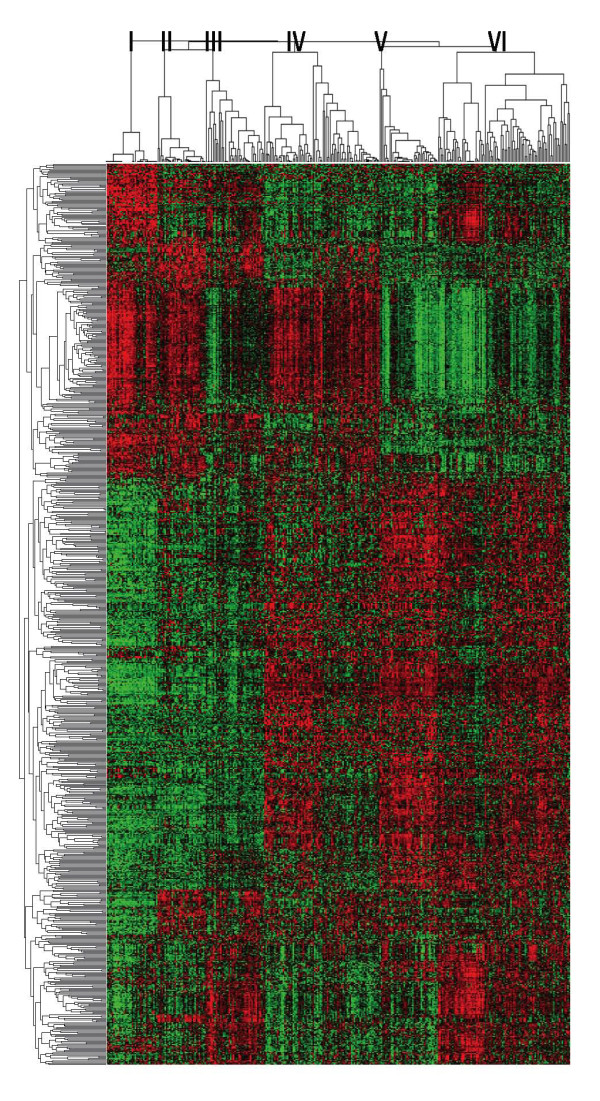
**Heat map of different molecular subtypes of breast cancer**. The dendrogram of the 783 classification probe-sets is shown on the left and 327 breast cancer samples clustered into six molecular subtypes are shown at the top. The approach used to generate these six molecular subtypes is detailed in the Additional file 2.

**Figure 2:**
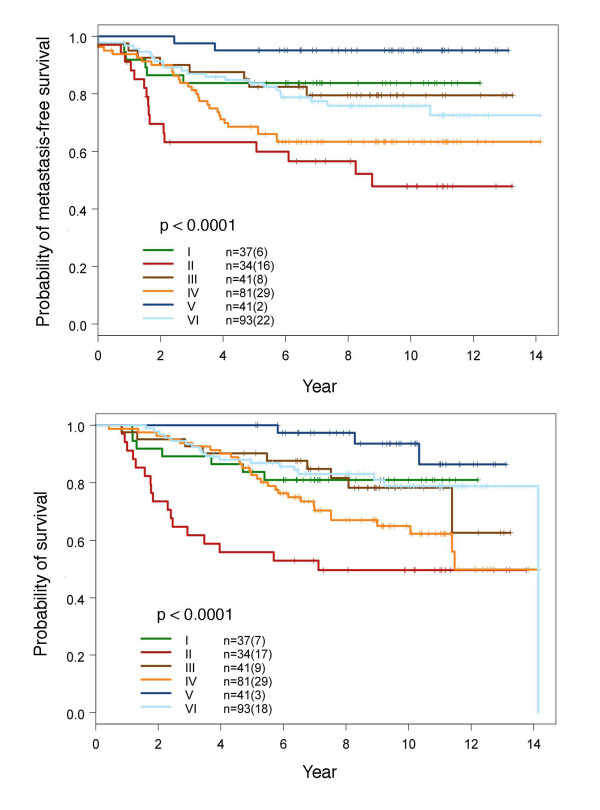
**Metastasis-free and overall survival curves of six different molecular subtypes of breast cancer**. Numbers in the parentheses are events. The *p *values were determined by log-rank test. The *p *values of log-rank test between any two of the six molecular subtypes are summarized in Table 3.

As shown in Figure 1, we classified breast cancer into six different molecular subtypes. The 783 probe-sets used for classification were grouped into 13 clusters enriched with genes associated with cell cycle/proliferation, cell movement, metabolism, and reproductive system development (Additional file 3, Figure S3). We then conducted statistical analysis between the molecular subtypes and various clinical parameters (Table 2). The results summarized in Table 2 show that by the T stage, smaller tumors dominated in subtypes V and VI, while larger tumors dominated in subtypes II, III and IV (p = 2 × 10^-5^). The majority of patients in subtypes IV, V and VI were positive for ER and PR (p = 6.3 × 10^-51 ^and 2.3 × 10^-18^, respectively). Interestingly, all subtype V breast cancers were positive for ER and PR and negative for HER2. In contrast, all subtype I breast cancers were negative for ER. Nearly all subtype II breast cancers were negative for ER (97%), and the majority had over-expression of HER2 (76.5%) (p = 9.1 × 10^-20^). Subtype III comprised breast cancers that had weaker ER and variable PR and HER2 expression (data not shown). Subtype IV had full range expression of HER2. Subtype II had the greatest propensity to develop distant metastases (47%) followed by subtypes IV (36%) and VI (24%), while subtype V was least likely to metastasize (5%) (p = 2.5 × 10^-5^). Figure 2 shows the survival curves of all six molecular subtypes. The statistical results of comparing survival outcomes between any two molecular subtypes are summarized in Table 3.

**Table 2:** Statistical comparison of clinical features among molecular subtypes.

	Subtype I		Subtype II		Subtype III		Subtype IV		Subtype V		Subtype VI		Fisher's exact test
	N = 37		N = 34		N = 41		N = 81		N = 41		N = 93		*p *value
**Age at diagnosis**													
< 50 yr	27	73.0%	16	47.1%	30	73.2%	54	66.7%	22	53.7%	54	58.1%	
>= 50 yr	10	27.0%	18	52.9%	11	26.8%	27	33.3%	19	46.3%	39	41.9%	0.08
**T stage**													
1	8	21.6%	4	11.8%	10	24.4%	16	19.8%	22	53.7%	41	44.1%	
2	28	75.7%	23	67.6%	20	48.8%	56	69.1%	17	41.5%	44	47.3%	
3	1	2.7%	5	14.7%	7	17.1%	5	6.2%	1	2.4%	7	7.5%	
4	0	0.0%	2	5.9%	4	9.8%	4	4.9%	1	2.4%	1	1.1%	**2.00E-05**
**N stage**													
0	20	54.1%	7	20.6%	16	39.0%	31	38.3%	20	48.8%	43	46.2%	
1	10	27.0%	10	29.4%	8	19.5%	25	30.9%	12	29.3%	22	23.7%	
2	4	10.8%	11	32.4%	11	26.8%	14	17.3%	7	17.1%	16	17.2%	
3	3	8.1%	6	17.6%	6	14.6%	11	13.6%	2	4.9%	12	12.9%	0.26
**Pos. Lym. Nodes**													
0	20	54.1%	6	17.6%	16	39.0%	31	38.3%	20	48.8%	43	46.2%	
1-3	10	27.0%	10	29.4%	8	19.5%	26	32.1%	12	29.3%	22	23.7%	
4-9	4	10.8%	11	32.4%	10	24.4%	13	16.0%	7	17.1%	16	17.2%	
> = 10	3	8.1%	5	14.7%	6	14.6%	9	11.1%	2	4.9%	12	12.9%	0.30
**M stage**													
0	36	97.3%	33	97.1%	40	97.6%	78	96.3%	41	100.0%	91	97.8%	
1	1	2.7%	1	2.9%	1	2.4%	3	3.7%	0	0.0%	2	2.2%	0.94
**TNM Stage**													
I	6	16.2%	2	5.9%	10	24.4%	9	11.1%	12	29.3%	28	30.1%	
II	23	62.2%	13	38.2%	11	26.8%	46	56.8%	18	43.9%	36	38.7%	
II	6	16.2%	18	52.9%	19	46.3%	23	28.4%	10	24.4%	27	29.0%	
IV	1	2.7%	1	2.9%	1	2.4%	3	3.7%	0	0.0%	2	2.2%	**7.60E-04**
**Nuclear Grade**													
1	1	2.7%	0	0.0%	2	4.9%	2	2.5%	9	22.0%	17	18.3%	
2	3	8.1%	1	2.9%	4	9.8%	11	13.6%	18	43.9%	38	40.9%	
3	30	81.1%	28	82.4%	33	80.5%	62	76.5%	10	24.4%	33	35.5%	**0**
ER*													
positive	0 (2)	0.0% (5%)	1 (6)	2.9% (20.6%)	10 (13)	24.4% (36%)	70 (66)	86.4% (90.4%)	41 (36)	100.0% (97.2%)	82 (75)	88.2% (91.5%)	
negative	37 (34))	100.0% (95%)	33 (23)	97.1% (79.4%)	31 (23)	75.6% (64%)	11 (7)	13.6% (9.6%)	0 (1)	0.0% (2.8%)	11 (7)	11.8% (8.5%)	**6.31E-51**
HER2*													
positive	4 (2)	10.8% (11.8%)	26 (9)	76.5% (81.8%)	18 (16)	43.9% (66.7%)	22 (11)	27.2% (33.3%)	0 (2)	0.0% (13.3%)	5 (7)	5.4% (15.9%)	
negative	33 (17)	89.2% (88.2%)	8 (2)	23.5% (18.2%)	23 (8)	56.1% (33.3%)	59 (22)	72.8% (66.7%)	41 (13)	100.0% (86.7%)	88 (37)	94.6% (84.1%)	**9.09E-20**
PR*													
positive	19 (9)	51.4% (25%)	14 (11)	41.2% (37.9%)	23 (16)	56.1% (44.4%)	73 (64)	90.1% (87.7%)	41 (34)	100.0% (91.9%)	88 (70)	94.6% (85.4%)	
negative	18 (27)	48.6% (75%)	20 (18)	58.8% (62.1%)	18 (20)	43.9% (55.6%)	8 (9)	9.9% (12.3%)	0 (3)	0.0% (8.1%)	5 (12)	5.4% (14.6%)	**2.26E-18**
**Local Relapse**													
No	31	83.8%	27	79.4%	39	95.1%	68	84.0%	34	82.9%	86	92.5%	
Yes	6	16.2%	4	11.8%	1	2.4%	8	9.9%	3	7.3%	6	6.5%	0.29
**Regional Relapse**													
No	32	86.5%	26	76.5%	37	90.2%	67	82.7%	36	87.8%	84	90.3%	
Yes	2	5.4%	5	14.7%	3	7.3%	6	7.4%	1	2.4%	8	8.6%	0.54
**Distant metastasis**													
No	31	83.8%	15	44.1%	33	80.5%	50	61.7%	39	95.1%	70	75.3%	
Yes	6	16.2%	16	47.1%	8	19.5%	29	35.8%	2	4.9%	22	23.7%	**2.51E-05**

*: Numbers in parentheses are results determined by immunohistochemistry. For HER2, 3+ was regarded as positive. *P *values for ER, HER2 and PR by IHC among all six molecular subtypes were 3 × 10^-36^, 2 × 10^-4 ^and 1.4 × 10^-14^, respectively.

**Table 3:** P values of log-rank test for metastasis-free and overall survival between any two molecular subtypes.

	*p *values of log rank test between molecular subtypes for metastasis-free survival				
	
	II	III	IV	V	VI
**I**	**0.0072**	0.7554	**0.0467**	0.0910	0.4455
**II**		**0.0081**	0.1431	**6.4E-06**	**0.0039**
**III**			0.0727	**0.04**	0.6582
**IV**				**0.0003**	0.0704
**V**					**0.0094**

	***p *values of log rank test between molecular subtypes for overall survival**				
	
	**II**	**III**	**IV**	**V**	**VI**

**I**	**0.0062**	0.9855	0.1702	0.0947	0.8725
**II**		**0.0066**	0.0521	**1.6E-05**	**0.0001**
**III**			0.1534	**0.0484**	0.6917
**IV**				**0.0009**	**0.0335**
**V**					0.0778

*P *values < 0.05 are shown in bold type.

### Molecular Characteristics and Validation of Breast Cancer Subtypes

**Figure 3:**
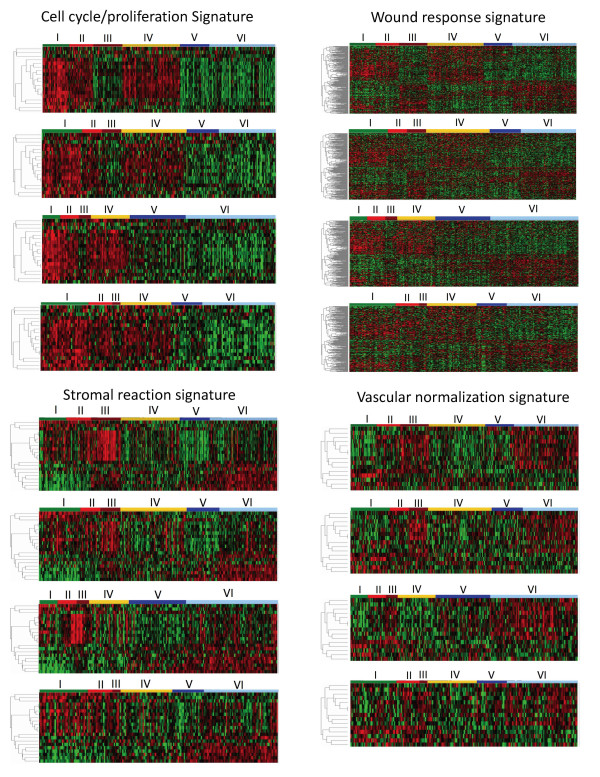
**Validation of molecular subtypes of breast cancer established in this study**. One-way hierarchical clustering analysis was performed on 327 samples in our dataset using genes associated with cell cycle/proliferation, wound-response [9], stromal reaction [21], and tumor vascular endothelial normalization [22,23]. Breast cancer samples were arranged according to their subtype as shown at the top of each panel. Dendrograms of signature genes are shown on the left. The identities of genes in all four dendrograms are listed in the Additional file 3, Figure S4. None of the genes used in this study were part of the 783 probe-sets used for molecular subtyping. The same gene clusters generated from our dataset were used to draw heat maps for the other three independent datasets. The heat maps from top to bottom for each signature were KFSYSCC, EMC [10], Uppsala [19], and TRANSBIG [20]. Each molecular subtype shared the same distinctive gene expression pattern among all four datasets. Subtypes I, II and IV showed increased expressions of cell cycle/proliferation genes. Subtypes I and II showed higher expression of stromal genes known to associate with poorer survival [21]. Subtypes III and VI had elevated expression of genes associated with vascular endothelial normalization. The concordance of differential gene expression for the six molecular subtypes between the KFSYSCC dataset and each of the other three independent datasets [10,19,20] was analyzed by Pearson correlation. The *p *value for each correlation coefficient was determined by comparing with null distribution based on 10,000 permutations of each independent dataset at subtype level. The Pearson correlation coefficient between the KFSYSCC dataset and that of EMC, Uppsala or TRANSBIG was 0.94, 0.92 or 0.87 for cell cycle/proliferation, 0.85, 0.84 or 0.78 for wound response, 0.94, 0.91 or 0.87 for stromal reaction, and 0.86, 0.86 or 0.83 for tumor vascular endothelial normalization. All *p *values were < 0.0001.

To demonstrate the biologically distinctive nature of six different subtypes of breast cancer, we studied the differential expressions of genes associated with cell cycle/proliferation, wound-response [9], stromal reaction [21] and vascular endothelial normalization [22,23] using one-way clustering analysis. Genes used in this study were not used for molecular subtyping. As shown in Figure 3, all six molecular subtypes demonstrated distinct gene expression characteristics. The dendrograms of the probe-sets and the probe-set IDs are summarized in Figure S4 of Additional file 3. For validation, we used our classifier genes with centroid analysis to determine molecular subtypes of breast cancer samples in three independent datasets [10,19,20]. We then compared differential gene expression patterns associated with cell cycle/proliferation, wound-response, stromal reaction and vascular endothelial normalization for the same molecular subtypes between our dataset and the other three independent datasets. The same molecular subtypes in all four datasets were shown to share the same differential gene expression patterns (Figure 3). For further validation, we employed a different approach. We selected five genes (*CAV1, DHFR, TYMS, VIM, ZEB1*) known to be associated with drug sensitivity and the epithelial-mesenchymal transition of breast cancer [25-29]. The intensity of expression of these genes was plotted according to molecular subtypes. Again, each molecular subtype shared the same unique molecular characteristics across all four datasets (Additional file 3, Figure S5).

### Correlation of Molecular Subtypes with Perou-Sørlie Intrinsic types

**Figure 4:**
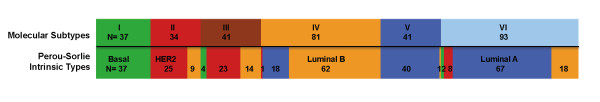
**Correlation of the molecular subtypes with the Perou-Sørlie intrinsic types**. The top row shows the color-coded molecular subtypes of 327 samples in our dataset, and the lower panel shows how the same cases on top were classified into the basal (green), HER2-overexpressing (red), luminal A (blue) and luminal B (brown) intrinsic types using the classification genes of Sørlie, et al. [2]. The results show both similarities and differences between the results of these two classification methods.

To study how the molecular subtypes of breast cancer used in this study are correlated with the Perou-Sørlie intrinsic types [1,2], we applied the classifier genes used by Perou-Sørlie [2] to our samples. As shown in Figure 4, there were both similarities and considerable differences between the two classification methods. A high degree of concordance was noted between our subtype I and the basal-like intrinsic type. When we applied our classification genes to the NKI dataset [24], wherein we also noticed an 89% concordance between our subtype I and the basal-like intrinsic type. The high degree of concordance was likely the result of the very distinctive features of this subtype of breast cancer.

Nevertheless, most of the Perou-Sørlie luminal A intrinsic type was divided into subtypes V and VI according to our classification genes (Figure 4). When we compared metastasis free survival between subtypes V and VI of the luminal A intrinsic type breast cancer patients in our cohorts, significantly better metastasis-free survival was observed for the subtype V patients comparing to the subtype VI (p = 0.025) (Additional file 3, Figure S6). There were no significant differences in disease severity (T stage p = 0.33, N stage p = 0.50, M stage p = 1, positive axillary lymph node number p = 0.50, and nuclear grade p = 1), however. The differentiation of subtypes V and VI breast cancer within the luminal A intrinsic type is therefore clinically significant. The distinction was further supported by the differential gene expression patterns for wound response and vascular endothelial normalization between these two subtypes of breast cancer (Figure 3).

The cases of HER2 over-expressing intrinsic type, they were divided into molecular subtypes II and III (Figure 4). We noted that cases of molecular subtype III cases classified as HER2 over-expressing intrinsic type expressed ER at a level higher than the subtype II and HER2 over-expressing intrinsic type (the average intensity of gene expression in logarithm to base 2 were 9.9 ± 0.96 vs. 8.6 ± 1.0, p < 0.0001). It appears that our molecular subtyping have discerned different subsets within the HER2 over-expressing intrinsic type. In this study, we did not find normal-breast like intrinsic type breast cancer in our cohorts.

### Differential Treatment Responses of Breast Cancer Molecular Subtypes

**Figure 5:**
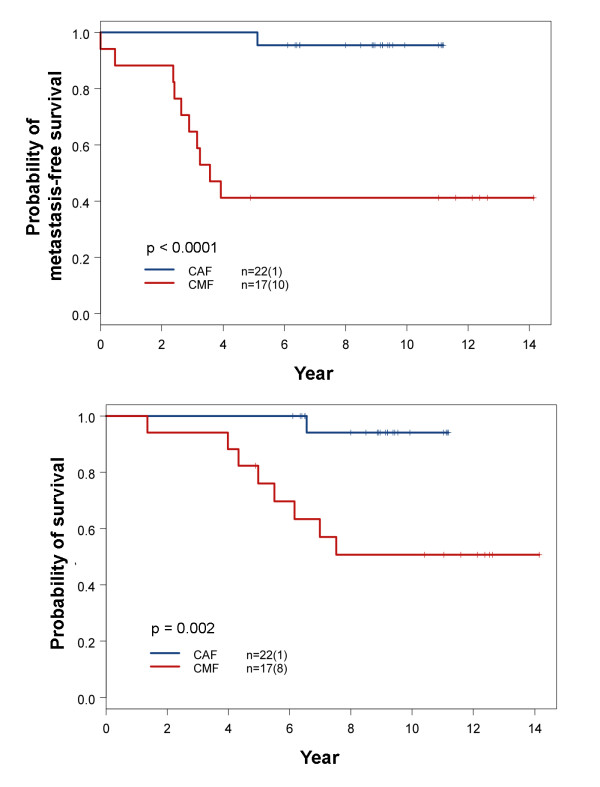
**Comparison of survival outcome between patients with molecular subtype IV breast cancer treated with CMF and CAF**. Detailed comparisons of pertinent clinical parameters between these two treatment groups are summarized in Table 4. The numbers in parentheses represent the number of events. *P *values were determined by log-rank test. The upper panel is metastasis-free survival curves and the lower panel is overall survival curves.

The breast cancer samples included in this study covered the period of transition of adjuvant chemotherapy regimen from CMF to CAF and to taxane-based regimens. These archival samples provided an opportunity to examine how breast cancer subtypes might have responded differentially to CMF and CAF regimens of adjuvant chemotherapy. The results of this study show that the change from methotrexate to doxorubicin had a major impact on the survival of patients with subtype IV breast cancer (Figure 5). None of the pertinent clinical factors between these two groups of patients showed a significant difference except for the N stage. The N stage was higher in the CAF group (Table 4). In spite of the higher N stage, significantly better metastasis-free and overall survival was observed for subtype IV patients treated with CAF than with CMF (Figure 5). For other molecular subtypes, we did not find significant differences in survival between groups receiving treatment with CAF or CMF (Additional file 1, Table S5). The small sample size did not allow us to draw firm conclusions regarding survival in molecular subtypes other than subtype IV.

**Table 4:** Statistical comparison of pertinent clinical parameters between subtype IV patients treated with CAF and CMF adjuvant chemotherapy.

	Molecular Subtype IV breast Cancer				
	
	CAF		CMF		*p *value*
	Patients (n = 22)		Patients (n = 17)		
**Age at diagnosis**					0.464
< 50 yr	15	68.2%	14	82.4%	
>= 50 yr	7	31.8%	3	17.6%	
**TNM Path T**					0.612
1	3	13.6%	2	11.8%	
2	19	86.4%	13	76.5%	
3	0	0.0%	1	5.9%	
4	0	0.0%	1	5.9%	
**TNM Path N**					0.047
0	9	40.9%	11	64.7%	
1	12	54.5%	3	17.6%	
2	1	4.5%	2	11.8%	
3	0	0.0%	1	5.9%	
**TNM Path M**					0.436
0	22	100.0%	16	94.1%	
1	0	0.0%	1	5.9%	
**Positive Lymph Nodes**					0.067
0	9	40.9%	11	64.7%	
1-3	12	54.5%	3	17.6%	
4-9	1	4.5%	2	11.8%	
**TNM Stage**					0.109
I	0	0.0%	2	11.8%	
II	21	95.5%	12	70.6%	
III	1	4.5%	2	11.8%	
IV	0	0.0%	1	5.9%	
**Nuclear Grade**					0.495
1	1	4.5%	0	0.0%	
2	2	9.1%	0	0.0%	
3	19	86.4%	17	100.0%	
**Post-op Radiation Rx**					1.000
No	14	63.6%	11	64.7%	
Yes	8	36.4%	6	35.3%	

*: *p *values were determined by Fisher's exact test.

There was no difference in follow-up duration between two groups (median 9.0 vs. 7.5 years, *p *= 0.90 by Wilcoxon rank sum test).

**Figure 6:**
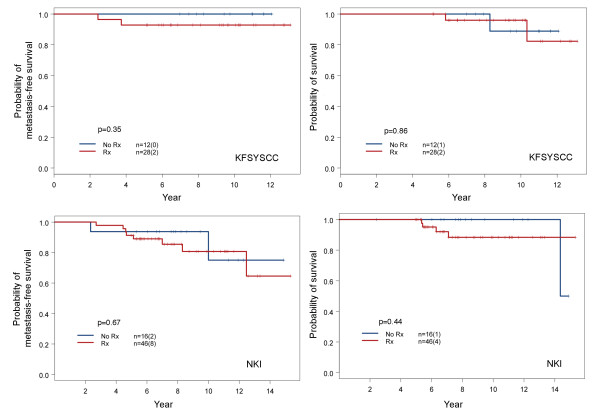
**Comparison of survival outcome between subtype V patients with and without adjuvant chemotherapy**. Comparisons of survival were conducted for patients in our dataset (upper panels) and the NKI dataset [24] (lower panels). The comparison of pertinent clinical parameters showed no differences between the two treatment groups from our KFSYSCC dataset (Table 5). Patients with subtype V breast cancer in the NKI database were identified using the classifier genes established in this study and centroid analysis. All NKI patients with N1 stage disease were selected for comparison. Tumor size distribution and the fraction of patients treated with hormonal therapy were not significantly different between the two treatment groups, with respective *p *values of 1.0 and 0.32 using Fisher's exact test. The NKI stage N0 patients were not included in this study because an overwhelming number did not receive adjuvant chemotherapy. Their inclusion would have caused an uneven distribution of disease severity. The results show that adjuvant chemotherapy did not provide survival benefit for patients with early stage subtype V breast cancer in either dataset.

A number of patients in our cohorts opted not to receive adjuvant chemotherapy even though it was indicated. This allowed us to study how the omission of adjuvant chemotherapy might have influenced patient survival among various subtypes of breast cancer. In a comparison of disease severity between those with and without adjuvant chemotherapy in each subtype, only patients with subtype V showed no significant difference (Table 5), thereby offering an interpretable comparison. As shown in Figure 6, the metastasis-free and overall survivals of subtype V were essentially the same between those who received adjuvant chemotherapy and those who did not. This suggests that adjuvant chemotherapy did not provide survival benefits to subtype V patients in the early stages; however, this would require further confirmation due to small sample size.

**Table 5:** Statistical comparison of pertinent clinical parameters between subtype V patients treated with and without adjuvant chemotherapy.

	Molecular Subtype V Breast Cancer				
	
	Adjuvant Rx		No-Adjuvant Rx		*p *values*
	Patient (n = 28)		Patient (n = 12)		
**Age at diagnosis**					
< 50 yr	16	57.1%	5	41.7%	0.49
>= 50 yr	12	42.9%	7	58.3%	
**TNM Path T**					
1	14	50.0%	8	66.7%	0.14
2	14	50.0%	3	25.0%	
3	0	0.0%	0	0.0%	
4	0	0.0%	1	8.3%	
**TNM Path N**					
0	13	46.4%	7	58.3%	0.86
1	8	28.6%	4	33.3%	
2	5	17.9%	1	8.3%	
3	2	7.1%	0	0.0%	
**TNM Path M**					
0	28	100.0%	12	100.0%	
**Positive Lymph Nodes**					
0	13	46.4%	7	58.3%	0.86
1-3	8	28.6%	4	33.3%	
4-9	5	17.9%	1	8.3%	
> = 10	2	7.1%	0	0.0%	
**TNM Stage**					
I	7	25.0%	6	50.0%	0.34
II	14	50.0%	4	33.3%	
III	7	25.0%	2	16.7%	
**Nuclear Grade**					
1	4	14.3%	5	41.7%	0.17
2	13	46.4%	4	33.3%	
3	8	28.6%	2	16.7%	
**Hormonal Therapy**					
No	3	10.7%	2	16.7%	0.63
Yes	25	89.3%	10	83.3%	
**Post-op Radiation Rx**					
No	20	71.4%	9	75.0%	1.00
Yes	8	28.6%	3	25.0%	
					

*: *p *values were determined by Fisher's exact test

There was no difference in follow-up duration between two groups (median 10.4 vs. 9.2 years, *p *= 0.17 by Wilcoxon rank sum test)

To seek further support of our finding, we studied patients from the NKI dataset with subtype V breast cancer of N1 stage [24]. This dataset includes treatment and survival outcome information, and many patients in this dataset did not receive adjuvant chemotherapy. The distribution of tumor size and fraction of patients treated with hormone therapy were not significantly different between those who received adjuvant chemotherapy and those who did not. The *p *values determined by Fisher's exact test were 0.32 and 1.0, respectively. Stage N0 patients were excluded because an overwhelming number were not treated with adjuvant chemotherapy. The results showed that there was no difference in survival between stage N1 subtype V patients treated with adjuvant chemotherapy and those who were not (Figure 6).

**Figure 7:**
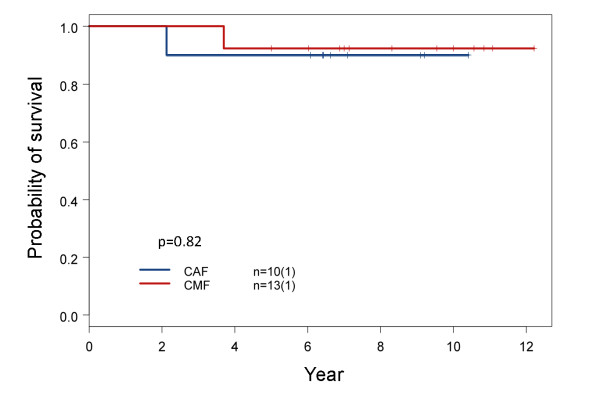
**Comparison of overall survival between subtype I patients treated with CAF and CMF adjuvant chemotherapy**. Clinical variables including age at diagnosis, TNM stages, positive lymph node number, nuclear grade, hormonal therapy and post-op radiation were compared between these two treatment groups. There were no significant differences (Additional file 1, Table S6). The results of this small sample size study are supported by a recent report on two large-scale clinical trials [32].

As mentioned earlier, subtype I breast cancer was essentially the same as the basal-like intrinsic type (Figure 4), and this subtype of breast cancer is known to be chemosensitive [30]. The five and ten year survival rates of patients with basal-like breast cancer who did not receive adjuvant chemotherapy were 64% and 44%, respectively [24]. This is consistent with the fact that basal-like breast cancer has aggressive clinical course and poor survival without adjuvant chemotherapy [31]. When we studied the survival of patients with subtype I breast cancer following CMF or CAF adjuvant chemotherapy, it was noticed that both groups had good long-term survival outcome based on the results from a limited number of patients (Figure 7). This suggests that subtype I breast cancer responds well to CMF adjuvant chemotherapy, and this finding is supported by a recent study of two large randomized clinical trials in which patients with node negative basal-like breast cancer were sensitive and responsive to CMF adjuvant chemotherapy and had good long-term survival following treatment [32]. Adjuvant chemotherapy is therefore critical for the long-term survival of patients with early stage subtype I breast cancer. The use of less toxic CMF could be as effective as CAF and deserves further study.

### Correlation of Molecular Subtypes with Risk of Recurrence Predicted by Oncotype™DX and MammaPrint^®^

**Figure 8:**
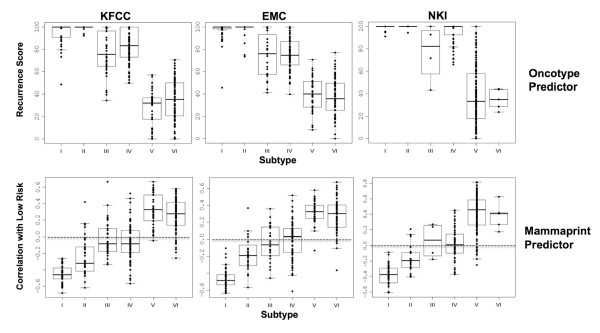
**Correlation between molecular subtypes and distant recurrence risks predicted by the Oncotype and MammaPrint predictor**. The three different datasets used in this study included ours (KFSYSCC), the EMC [10] and the NKI [24]. The number of cases in each subtype for the KFSYSCC, EMC, and NKI datasets were 37, 49, and 10 for subtype I; 34, 24, and 18 for subtype II; 41, 24, and 4 for subtype III; 81, 80, and 52 for subtype IV; 41, 39 and 172 for subtype V; and 93, 70 and 9 for subtype VI, respectively. The method used to score the risk of distant recurrence is detailed in Additional File 2. For prediction of recurrence risk by genes of the Oncotype predictor, a higher score represents a higher risk of recurrence. The negative correlation scores predicted by the MammaPrint predictor shown on the y axis represent higher risk of distant recurrence. A score of <0 can be defined as high risk for recurrence and a score of = or >0 as low risk.

Oncotype and MammaPrint predictors are used to predict the risk of distant recurrence in breast cancer patients for the optimization of treatment [12,13,33]. To learn how the groups with varying levels of the predicted risk are correlated with molecular subtypes of breast cancer, we conducted a study on patients in our dataset and the other two independent datasets [10,24]. We determined molecular subtypes and the scores of relative risk of distant recurrence. The results, summarized in Figure 8, reveal that patients with a high risk of distant recurrence according to the genes of Oncotype predictor included both subtypes I (basal-like) and II (HER2 over-expressing). Low risk cases were mostly subtypes V and VI, while most intermediate risk cases were subtypes III and IV. The high-risk cases predicted by the genes of MammaPrint included most of subtype I and many of subtypes II, III and IV, with low risk cases limited to subtypes V and VI (Figure 8). The results were consistent across all three datasets; thus, breast cancer within the same predicted risk group is heterogeneous according to molecular subtype. Patients within the same risk group may require different therapeutic approaches for better survival outcome.

## Discussion

This paper reports the results of a gene expression profiling study in which breast cancer samples were collected over a fourteen year period (1991-2004). The study was prompted by the fact that the current clinical application of microarray-based prediction for the customization of breast cancer treatment is restricted to predicting the risk of distant recurrence (e.g. MammaPrint). The clinical application of molecular subtypes based on high dimensional gene expression profiles reflecting the intrinsic biology of breast cancer remains unrealized. One reason for this lack of progress has to do with the absence of preliminary reports on how microarray-based molecular subtypes could be correlated with clinical outcomes resulting from various treatments of breast cancer. The long duration covered by our study enabled us to investigate how a change in adjuvant chemotherapy regimens might have influenced the survival outcome of patients with various molecular subtypes of breast cancer.

It is known that different designs of microarray platforms and methods of preparing mRNA targets could lead to less-than-perfect direct cross-platform application of classifier genes [34-36]. To establish a reliable and robust methodology for molecular subtyping for this study and future clinical application, we developed and validated a platform-specific method. Our classification method is based on the assumptions that genes with expression levels quantitatively correlated with the expression of pivotal genes play important roles in determining the biology and clinical behavior of breast cancer, and that genes with a low kurtosis score and more than one peak distribution could be robust for classification. Six different molecular subtypes showing distinctive molecular characteristics and clinical behavior were identified.

For validation, we applied our classifier genes to three independent datasets [10,19,20] and examined whether each molecular subtype in the different datasets shared the same unique gene expression patterns associated with cell cycle proliferation, wound response, stromal reaction and vascular endothelial normalization of tumors, in each of the datasets. We found that the same subtype shared the same unique gene expression patterns across all datasets (Figure 3). The selection of this validation approach enabled us to avoid heterogeneity in clinical outcomes associated with various approaches of treatment and patient selection criteria used in different gene expression profiling datasets.

Our method of breast cancer molecular subtyping was also validated by the consistent correlations between the molecular subtypes generated by our classifier genes and the risk of distant recurrence predicted by the genes used in the Oncotype and MammaPrint predictors among three different datasets (Figure 8). We noted that a disproportionally low number of cases of subtype VI breast cancer cases in the NKI dataset. This was likely due to the cross-platform application of our classifier genes to the NKI dataset. We were unable to reliably differentiate subtypes V and VI breast cancer in the NKI dataset. However, this failure did not influence the conclusions drawn from this study, because both subtypes V and VI were predicted as low risk for distant recurrence in all three datasets (Figure 8). The results of this study reveal that the same risk group predicted by the genes of Oncotype or MammaPrint predictor comprises different molecular subtypes of breast cancer (Figure 8).

The present study suggests that different molecular subtypes of breast cancer within a group sharing the same predicted risk of distant recurrence could benefit from different treatments. For instance, the groups predicted as high-risk by the genes of Oncotype predictor include subtypes I, II, III and IV. Nevertheless, subtype I breast cancer was chemosensitive and could respond equally well to CMF and CAF in the early stages (Figure 7). Despite of the small sample size, this conclusion is supported by a recent study of two large-scale clinical trials showing that triple negative basal-like breast cancer responds well to the treatment of CMF adjuvant chemotherapy [32]. In contrast, subtype IV breast cancer appeared resistant to methotrexate and sensitive to a chemotherapy regimen containing anthracycline (Figure 5). We also noticed good survival outcome in subtype IV breast cancer patients who had an over-expression of HER2 and were treated with CAF without trastuzumab. It appears that subtype IV breast cancer patients with over-expression of HER2 could be adequately treated with chemotherapy regimen containing anthracycline without costly trastuzumab. In contrast, subtype II breast cancer patients with over-expression of HER2 had the worst survival despite adjuvant chemotherapy (Figure 2). Patients of this subtype may benefit most from trastuzumab therapy or other tyrosine kinase receptor inhibitors.

Patients in the group predicted as having low risk for distant recurrence were mostly classified as subtype V or VI (Figure 8). The results of this study show that subtype V is a unique subset of the Perou-Sørlie luminal A intrinsic type (Table 2, Figure 4 and Figure 6). Early stage subtype V patients had excellent survival outcome even without adjuvant chemotherapy (Figure 6). This finding was confirmed by comparing the survival of subtype V patients from the NKI dataset who had received adjuvant chemotherapy and those who had not (Figure 6). The absence of benefit from adjuvant chemotherapy for subtype V patients was also supported by a recently study in which most stage II-III breast cancer patients predicted by MammaPrint as having a low risk of recurrence did not respond to neoadjuvant chemotherapy [37].

Patients with subtype VI had a higher risk of developing distant metastasis than those with subtype V breast cancer (Figure 2 and Table 3). Our study also showed that subtypes V and VI have very different molecular characteristics. For instance, like subtype III, subtype VI has a strong vascular endothelial normalization signature, but this is not the case for subtype V (Figure 3). Subtype VI has a significantly higher expression of genes characteristic of epithelial-mesenchymal transition (e.g. *TWIST2, SNAI2, ZEB2, VIM*) than subtype V (Additional file 3, Figure S7). For this reason, adjuvant chemotherapy may not be safely omitted from the treatment of patients with subtype VI. Differentiation between these two molecular subtypes can be clinically important. For the reasons discussed above, treatment of breast cancer patients in groups with the same risk of recurrence requires further customization, according to the respective molecular subtype of the disease.

**Figure 9:**
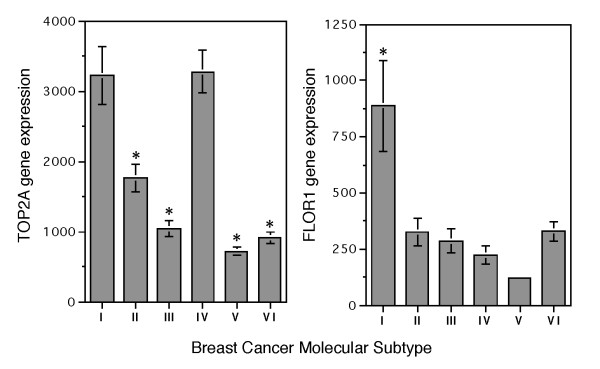
**Average expression intensity of *TOP2A *and *FLOR1 *genes in different molecular subtypes of breast cancer**. All patients (n = 327) in our dataset were included in the study. The average expression of each gene is shown as mean ± SEM. Student t test was conducted between subtype IV and other subtypes following logarithmic transformation of expression intensities to base of 2. *TOP2A *expression of subtype IV was significantly higher than subtype II, III, V and VI with *p *values of < 0.0001 (*). There was no significant difference between subtype IV and I. For expression of FLOR1, subtype IV was significantly lower than subtypes I with *p *< 0.0001(*). The number of samples in each subtype is available in Table 2.

Identification of subtype IV breast cancer in the present study may have provided answer to an ongoing debate regarding the presence and identification of a subset of breast cancer showing excellent response to anthracycline [14-16]. According to the results of our study, only subtype IV breast cancer showed a significantly different response to treatment with CAF or CMF adjuvant chemotherapy (Figure 5). *TOP2A *is known as a target for anthracyclines. Breast cancer with increased *TOP2A *expression has been reported to be more sensitive to anthracycline [15,38]. Both subtypes I and IV breast cancer in our study indeed had the highest *TOP2A *expression among the six molecular subtypes (Figure 9). With regard to drug sensitivity to methotrexate, it is known that an increase in the expression of *DHFR *and reduced expression of genes involved in methotrexate transport (*SLC19A1 *and *FOLR1*) and retention (*FPGS*) can contribute to resistance to methotrexate [39]. Statistical comparisons of the expression of the genes between subtype I and IV showed significant differences in the expression of folate receptor alpha (*FOLR1*) (Figure 9) with no differences for *SLC19A1, FPGS *or *DHFR*. The reduced expression of *FOLR1 *might have contributed to the poor response of subtype IV breast cancer to the methotrexate-containing CMF regimen. Therefore, treating subtype IV breast cancer with anthracycline-containing regimen is critical.

## Conclusions

Results of this study indicate that breast cancer can be classified into six different molecular subtypes using Affymetrix U133 plus 2.0 GeneChip™. These six subtypes show both significant similarities and differences with the Perou-Sørlie intrinsic types, and have distinctive molecular and clinical characteristics. The correlation between molecular subtypes and responses to treatments demonstrates that microarray-based molecular subtyping in conjunction with pertinent clinical data can be used for the customization and optimization of breast cancer treatment. Carefully designed prospective clinical trials will be needed to confirm such clinical utility.

## Abbreviations

CAF: cyclophosphamide-Adriamycin-fluorouracil; CMF: cyclophosphamide-methotrexate-fluorouracil; DHFR: dihydrofolate reductase; ER: estrogen receptor (alpha); FOLR1: folate receptor alpha; FPGS: folypolyglutamate synthase; HER2: human epidermal growth factor receptor 2; IHC: immunohistochemistry; KFSYSCC: Koo Foundation SYS Cancer Center; NKI: the Netherlands Cancer Institute; PR: progesterone receptor; SLC19A1: folate transporter 1; TNM: TNM Classification of Malignant Tumors, T-tumor size, N-lymph nodes, M-distant metastasis; TOP2A: DNA topoisomerase 2-alpha.

## Competing interests

The authors declare that they have no competing interests.

## Authors' contributions

KJK and ATH contributed to concept design and manuscript writing. KJK, KMC, HCH and ATH contributed to data collection, assembly, analyses, and interpretation. All authors have read and approved the final manuscript.

## Pre-publication history

The pre-publication history for this paper can be accessed here:

http://www.biomedcentral.com/1471-2407/11/143/prepub

## Supplementary Material

Additional file 1 **Supplemental Tables S1-S6**. **This set of additional files includes the following supplemental tables**. Table S1 Twenty three pivotal genes used to identify probe-sets showing linear or quadratical correlation. Table S2 List of 783 probe-sets used for molecular subtyping of breast cancer and their gene cluster designations are shown in Figure S3. Table S3 Thirty probe-sets representing cell-cycle and proliferation genes Table S4 Probe-set IDs and genes from the OncotypeDX and MammaPrint predictors. They were used to determine recurrence risk scores. Table S5 Survival differences between patients treated with CMF and CAF adjuvant chemotherapy in each molecular subtype of breast cancer. Table S6 Statistical comparison of pertinent clinical parameters between subtype I patients treated with CAF and CMF adjuvant chemotherapy. Click here for file

Additional file 2 **Supplemental Methodology**. Methodology includes four sections: I) procedures for selection of classification probe-sets and molecular subtyping by two steps k-means clustering analysis; II) determination of cut-point values for estrogen receptor (ER), progesterone receptor (PR) and HER2; III) scoring relative risk of distant recurrence using genes of the OncotypeDX and MammaPrint predictor; and IV) statistical comparison for concordance of differential gene expression patterns among six breast cancer subtypes between KFSYSCC dataset and public datasets from EMC (ref. [10]), Uppsala (ref. [19]), and TRANSBIG (ref. [20]). Click here for file

Additional file 3 **Supplemental Figures S1-S7**. **This set of additional files includes the following six supplemental figures**. Figure S1 Cut-points to determine positivity of ER, PR and HER2. Figure S2 Correlation studies between immunohistochemistry and gene expression results for ER, PR and HER2 statuses. Figure S3 Functional annotation of gene clusters for breast cancer molecular subtyping. Figure S4 Dendrograms of genes associated with cell cycle/proliferation, stromal reaction, wound response and vascular endothelial normalization for characterizing breast cancer molecular subtypes. Figure S5 Differential expression of the selected genes by breast cancer molecular subtypes in different datasets. Figure S6 Comparison of metastasis-free survival between Subtypes V and VI breast cancer patients classified as Perou-Sørlie luminal A intrinsic type in patients of the present study. Figure S7 Differential expression of genes associated with epithelial-mesenchymal transition among breast cancer molecular subtypes of the present study. Click here for file
