# Supplementary material for: Correlation of microarray-based breast cancer molecular subtypes and clinical outcomes: implications for treatment optimization
Source: BMC Cancer. 2011 Apr 18;11:143. doi: 10.1186/1471-2407-11-143 (PMC3094326; doi:10.1186/1471-2407-11-143)
Supplement: Additional file 1 — Supplemental Tables S1-S6. This set of additional files includes the following supplemental tables. Table S1 Twenty three pivotal genes used to identify probe-sets showing linear or quadratical correlation. Table S2 List of 783 probe-sets used for molecular subtyping of breast cancer and their gene cluster designations are shown in Figure S3. Table S3 Thirty probe-sets representing cell-cycle and proliferation genes Table S4 Probe-set IDs and genes from the OncotypeDX and MammaPrint predictors. They were used to determine recurrence risk scores. Table S5 Survival differences between patients treated with CMF and CAF adjuvant chemotherapy in each molecular subtype of breast cancer. Table S6 Statistical comparison of pertinent clinical parameters between subtype I patients treated with CAF and CMF adjuvant chemotherapy. [file 1471-2407-11-143-S1.PDF]

Additional File 1

Supplemental Tables

for

Correlation of Microarray-based Breast Cancer Molecular Subtypes and Clinical Outcomes: Implications for Treatment Optimization

Kuo-Jang Kao, Kai-Ming Chang, Hui-Chi Hsu and Andrew T. Huang

Tables S1-S6

Table S1 Twenty three pivotal genes used to identify probe-sets showing linear or quadratical correlation.

Table S2 List of 783 probe-sets used for molecular subtyping of breast cancer and their gene cluster designation as shown in Figure S3.

Table S3 Thirty probe-sets representing cell-cycle and proliferation genes

Table S4 Probe-set IDs and genes from the OncotypeDX and MammaPrint predictors.

Table S5 Survival differences between patients treated with CMF and CAF adjuvant chemotherapy in each molecular subtype of breast cancer.

Table S6 Statistical comparison of pertinent clinical parameters between subtype I patients treated with CAF and CMF adjuvant chemotherapy.

Table S1. Twenty three pivotal genes used to identify probe-sets showing linear or quadratical correlation. The 23 pivotal genes were selected from the literature for their known involvement in the tumorigenesis and biology of breast cancer.

Gene Symbol	Probe-set	References
BIRC5	202094_at	Clin Cancer Res 6:127-34, 2000; Anticancer Res 22:1839-43, 2002; Clin Cancer Res 14:2681-9, 2008.
BRCA1	204531_s_at	Am J Hum Genet 52:702-10, 1993; Lancet 341:1101-2, 1993; N Engl J Med 334:137-42, 1996.
CD24	208650_s_at	Cancer Lett 143:87-94, 1999; Clin Cancer Res 11:1154-9, 2005; Breast Cancer Res 8:R59, 2006.
CEACAM6	203757_s_at	Clin Cancer Res 12:4773-83, 2006;Clin Cancer Res 14:405-11, 2008.
CENPF	207828_s_at	Int J Cancer. 120:1434-43, 2007.
CLDN1	218182_s_at	Breast Cancer Res 7:R296-305, 2005; Int J Mol Med. 20:139-43, 2007.
EGFR	201984_s_at	J Clin Oncol . 19:3376-84, 2001; BMC Genomics 31;8:258, 2007; Cancer Res 65:11018-25, 2005.
ERBB2	216836_s_at	Science 135:277-282, 1982; J Clin Oncol 19:3376-84, 2001; Human Mol Genetics, 12:3245–3258, 2003
ESR1	205225_at	Ann Surg 182:342-351, 1975; Cancer Research (suppl.) 46:4256s-4264s, 1986; Breast Cancer Res Treat 3:103-10,1983.
FGFR2	203638_s_at	Carcinogenesis 30:269-74, 2009; Nature 447:1087-93, 2007.
FOXA1	204667_at	Int J Cancer 120:1013-22, 2007; Clin Cancer Res 13:4415-21, 2007; Biochem Biophys Res Commun 365:711-7, 2008.
FOXC1	1553613_s_at	Proc Natl Acad Sci, U S A. 105:14076-81, 2008; J. R. Soc. Interface 3:367-381, 2006.
FOXO1	202723_s_at	Cell 117:421–426, 2004; Oncogene 24:7410–7425, 2005.
GRB7	210761_s_at	EMBO J 13:1331-40, 1994.
HMGA1	206074_s_at	Clinl Cancer Res 10:7637-7644, 2004; Oncogene 23:777-85, 2004; Mol Cell Biol 23:2225-38, 2003
MAP3K1	225927_at	Carcinogenesis 30:269-74, 2009; J Biol Chem 279:33085-92, 2004.
MKI67	212022_s_at	Eur J Cancer 29A:1501-2, 1993; Br J Cancer 96:1504-13, 2007; Nature 447:1087-93, 2007.
PGR	208305_at	Breast Cancer Res Treat 3:103-10, 1983; Ann N Y Acad Sci 286:90-100, 1977.
PRC1	218009_s_at	Cancer Sci 98:174-81, 2007;Exp Mol Med 40:345-53, 2008.
PRKAA1	225984_at	Crit Rev Oncol Hematol 67:1-7, 2008.
PTEN	225363_at	Science 275:1943-7, 1997; Oncogene 17:123-7,1998; Mod Pathol 14:672-6, 2001.
TOP2A	201292_at	Br J Cancer 95:1334-41, 2006; Mod Pathol 13:542-7, 2000.
TOX3	214774_x_at	Breast Cancer Res 11:R10, 2009; Nature 447:1087-93,2007.

Table S2 a-to-j. List of 783 probe-sets used for molecular subtyping of breast cancer and their gene cluster designation as shown in Figure S3.

Table S2a

No.	Probeset ID	Gene cluster	Gene Symbol	No.	Probeset ID	Gene cluster	Gene Symbol
1	228278_at	1	*NFIX*	41	225431_x_at	1	*PM20D2*
2	229341_at	1	*TFCP2L1*	42	230291_s_at	1	*NFIB*
3	205375_at	1	*MDFI*	43	230791_at	1	
4	226029_at	1	*VANGL2*	44	202965_s_at	1	*CAPN6*
5	213432_at	1	*MUC5B*	45	209815_at	1	*PTCH1*
6	220196_at	1	*MUC16*	46	224400_s_at	1	*CHST9*
7	204724_s_at	1	*COL9A3*	47	208399_s_at	1	*EDN3*
8	221029_s_at	1	*WNT5B*	48	209270_at	1	*LAMB3*
9	217728_at	1	*S100A6*	49	219932_at	1	*SLC27A6*
10	222784_at	1	*SMOC1*	50	202036_s_at	1	*SFRP1*
11	226611_s_at	1	*PRR6*	51	202037_s_at	1	*SFRP1*
12	238759_at	1	*CCDC88A*	52	209842_at	1	*SOX10*
13	239233_at	1	*CCDC88A*	53	212730_at	1	*DMN*
14	223075_s_at	1	*C9orf58*	54	1553977_a_at	1	*CYP39A1*
15	1552532_a_at	1	*ATP6V1C2*	55	213456_at	1	*SOSTDC1*
16	1553989_a_at	1	*ATP6V1C2*	56	211002_s_at	1	*TRIM29*
17	200824_at	1	*GSTP1*	57	212236_x_at	1	*KRT17*
18	201984_s_at	1	*EGFR*	58	213680_at	1	*KRT6B*
19	203637_s_at	1	*MID1*	59	219545_at	1	*KCTD14*
20	242671_at	1		60	204745_x_at	1	*MT1G*
21	223062_s_at	1	*PSAT1*	61	206461_x_at	1	*MT1H / MT1P2*
22	201030_x_at	1	*LDHB*	62	211456_x_at	1	*MT1P2*
23	213564_x_at	1	*LDHB*	63	212859_x_at	1	*MT1E*
24	214581_x_at	1	*TNFRSF21*	64	216336_x_at	1	*MT1M*
25	231034_s_at	1	*NHSL1*	65	219936_s_at	1	*GPR87*
26	232105_at	1		66	203021_at	1	*SLPI*
27	218796_at	1	*FERMT1*	67	203510_at	1	*MET*
28	226907_at	1	*PPP1R14C*	68	227238_at	1	*MUC15*
29	205350_at	1	*CRABP1*	69	213711_at	1	*KRT81*
30	221016_s_at	1	*TCF7L1*	70	221609_s_at	1	*WNT6*
31	204304_s_at	1	*PROM1*	71	214614_at	2	*MNX1*
32	215729_s_at	1	*VGLL1*	72	233446_at	2	*ONECUT2*
33	206373_at	1	*ZIC1*	73	239911_at	2	*ONECUT2*
34	233320_at	1	*TCAM1*	74	205766_at	2	*TCAP*
35	213122_at	1	*TSPYL5*	75	224447_s_at	2	*C17orf37*
36	220624_s_at	1	*ELF5*	76	210761_s_at	2	*GRB7*
37	228214_at	1		77	216836_s_at	2	*ERBB2*
38	226425_at	1	*CLIP4*	78	228066_at	2	
39	1553613_s_at	1	*FOXC1*	79	1565595_at	2	
40	225421_at	1	*PM20D2*	80	1569940_at	2	*SLC6A16*

Table S2-b List of 783 probe-sets used for classification of breast cancer

No.	Probeset ID	Gene cluster	Gene Symbol	No.	Probeset ID	Gene cluster	Gene Symbol
81	219271_at	2	*GALNT14*	121	222848_at	3	*CENPK*
82	209803_s_at	2	*PHLDA2*	122	214710_s_at	3	*CCNB1*
83	205916_at	2	*S100A7*	123	228729_at	3	*CCNB1*
84	202917_s_at	2	*S100A8*	124	228559_at	3	*CENPN*
85	203535_at	2	*S100A9*	125	219105_x_at	3	*ORC6L*
86	225165_at	2	*PPP1R1B*	126	219493_at	3	*SHCBP1*
87	234310_s_at	2	*SUSD2*	127	201710_at	3	*MYBL2*
88	204818_at	2	*HSD17B2*	128	223570_at	3	*MCM10*
89	228523_at	2	*NANOS1*	129	228273_at	3	
90	206827_s_at	2	*TRPV6*	130	204162_at	3	*NDC80*
91	1554712_a_at	2	*GLYATL2*	131	225834_at	3	*FAM72A / 72B*
92	243585_at	2	*ATP13A5*	132	228069_at	3	*FAM54A*
93	242350_s_at	2		133	204033_at	3	*TRIP13*
94	242414_at	2	*QPRT*	134	205339_at	3	*STIL*
95	220414_at	2	*CALML5*	135	204822_at	3	*TTK*
96	216641_s_at	2	*LAD1*	136	202870_s_at	3	*CDC20*
97	219010_at	2	*C1orf106*	137	209408_at	3	*KIF2C*
98	225316_at	2	*MFSD2*	138	202095_s_at	3	*BIRC5*
99	207076_s_at	2	*ASS1*	139	202094_at	3	*BIRC5*
100	213094_at	2	*GPR126*	140	204318_s_at	3	*GTSE1*
101	202411_at	3	*IFI27*	141	218662_s_at	3	*NCAPG*
102	219211_at	3	*USP18*	142	203554_x_at	3	*PTTG1*
103	204475_at	3	*MMP1*	143	218755_at	3	*KIF20A*
104	205568_at	3	*AQP9*	144	207746_at	3	*POLQ*
105	207469_s_at	3	*PIR*	145	206102_at	3	*GINS1*
106	240788_at	3		146	204092_s_at	3	*AURKA*
107	204058_at	3	*ME1*	147	208079_s_at	3	*AURKA*
108	204059_s_at	3	*ME1*	148	219306_at	3	*KIF15*
109	218883_s_at	3	*MLF1IP*	149	202954_at	3	*UBE2C*
110	229305_at	3	*MLF1IP*	150	210052_s_at	3	*TPX2*
111	228033_at	3	*E2F7*	151	218355_at	3	*KIF4A / KIF4B*
112	204886_at	3	*PLK4*	152	222077_s_at	3	*RACGAP1*
113	204887_s_at	3	*PLK4*	153	38158_at	3	*ESPL1*
114	205034_at	3	*CCNE2*	154	214804_at	3	
115	204026_s_at	3	*ZWINT*	155	223381_at	3	*NUF2*
116	203214_x_at	3	*CDC2*	156	229538_s_at	3	*IQGAP3*
117	210559_s_at	3	*CDC2*	157	219148_at	3	*PBK*
118	225655_at	3	*UHRF1*	158	204170_s_at	3	*CKS2*
119	229551_x_at	3	*ZNF367*	159	204709_s_at	3	*KIF23*
120	211713_x_at	3	*KIAA0101*	160	203362_s_at	3	*MAD2L1*

Table S2-c List of 783 probe-sets used for classification of breast cancer

No.	Probeset ID	Gene cluster	Gene Symbol	No.	Probeset ID	Gene cluster	Gene Symbol
161	213226_at	3	*CCNA2*	201	215623_x_at	3	*SMC4*
162	222958_s_at	3	*DEPDC1*	202	233436_at	3	*MTBP*
163	235545_at	3	*DEPDC1*	203	240247_at	3	
164	204444_at	3	*KIF11*	204	244427_at	3	*KIF23*
165	218542_at	3	*CEP55*	205	203744_at	3	*HMGB3*
166	218009_s_at	3	*PRC1*	206	228401_at	3	
167	203755_at	3	*BUB1B*	207	218782_s_at	3	*ATAD2*
168	218039_at	3	*NUSAP1*	208	222740_at	3	*ATAD2*
169	228323_at	3	*CASC5*	209	219215_s_at	3	*SLC39A4*
170	204825_at	3	*MELK*	210	228262_at	3	*MAP7D2*
171	206364_at	3	*KIF14*	211	200935_at	3	*CALR*
172	219918_s_at	3	*ASPM*	212	222222_s_at	3	*HOMER3*
173	202705_at	3	*CCNB2*	213	208502_s_at	3	*PITX1*
174	203764_at	3	*DLG7*	214	209587_at	3	*PITX1*
175	225687_at	3	*FAM83D*	215	223530_at	3	*TDRKH*
176	1552619_a_at	3	*ANLN*	216	201897_s_at	3	*CKS1B*
177	222608_s_at	3	*ANLN*	217	232065_x_at	3	*CENPL*
178	212022_s_at	3	*MKI67*	218	219032_x_at	4	*OPN3*
179	1555758_a_at	3	*CDKN3*	219	217388_s_at	4	*KYNU*
180	209714_s_at	3	*CDKN3*	220	205306_x_at	4	*KMO*
181	207165_at	3	*HMMR*	221	211138_s_at	4	*KMO*
182	209709_s_at	3	*HMMR*	222	203059_s_at	4	*PAPSS2*
183	202240_at	3	*PLK1*	223	1558034_s_at	4	*CP*
184	202107_s_at	3	*MCM2*	224	204846_at	4	*CP*
185	204146_at	3	*RAD51AP1*	225	228143_at	4	*CP*
186	223307_at	3	*CDCA3*	226	226021_at	4	*RDH10*
187	235609_at	3		227	222326_at	4	
188	203145_at	3	*SPAG5*	228	203708_at	4	*PDE4B*
189	223229_at	3	*UBE2T*	229	211302_s_at	4	*PDE4B*
190	218585_s_at	3	*DTL*	230	202728_s_at	4	*LTBP1*
191	222680_s_at	3	*DTL*	231	207332_s_at	4	*TFRC*
192	201292_at	3	*TOP2A*	232	222939_s_at	4	*SLC16A10*
193	207828_s_at	3	*CENPF*	233	206558_at	4	*SIM2*
194	209172_s_at	3	*CENPF*	234	203304_at	4	*BAMBI*
195	204558_at	3	*RAD54L*	235	208650_s_at	4	*CD24*
196	219787_s_at	3	*ECT2*	236	206023_at	4	*NMU*
197	225777_at	3	*C9orf140*	237	214595_at	4	*KCNG1*
198	202779_s_at	3	*LOC731049*	238	218051_s_at	4	*NT5DC2*
199	203276_at	3	*LMNB1*	239	226346_at	4	*MEX3A*
200	209680_s_at	3	*KIFC1*	240	227512_at	4	*MEX3A*

Table S2-d List of 783 probe-sets used for classification of breast cancer

No.	Probeset ID	Gene cluster	Gene Symbol	No.	Probeset ID	Gene cluster	Gene Symbol
241	206074_s_at	4	*HMGA1*	281	204364_s_at	7	*REEP1*
242	201195_s_at	4	*SLC7A5*	282	204365_s_at	7	*REEP1*
243	205282_at	4	*LRP8*	283	219686_at	7	*STK32B*
244	204695_at	4	*CDC25A*	284	243806_at	7	
245	217755_at	4	*HN1*	285	227764_at	7	*LYPD6*
246	231195_at	4	*KLRG2*	286	202769_at	7	*CCNG2*
247	233691_at	4		287	202770_s_at	7	*CCNG2*
248	208767_s_at	4	*LAPTM4B*	288	211559_s_at	7	*CCNG2*
249	208029_s_at	4	*LAPTM4B*	289	211538_s_at	7	*HSPA2*
250	214039_s_at	4	*LAPTM4B*	290	219682_s_at	7	*TBX3*
251	203560_at	4	*GGH*	291	229576_s_at	7	*TBX3*
252	212142_at	4	*MCM4*	292	204573_at	7	*CROT*
253	222037_at	4	*MCM4*	293	209522_s_at	7	*CRAT*
254	235003_at	5	*UHMK1*	294	203343_at	7	*UGDH*
255	222158_s_at	5	*C1orf121*	295	224989_at	7	
256	239392_s_at	5		296	210319_x_at	7	*MSX2*
257	210057_at	5	*SMG1*	297	239066_at	7	
258	235167_at	5	*DKFZp547E087*	298	206457_s_at	7	*DIO1*
259	241755_at	5	*UQCRC2*	299	202862_at	7	*FAH*
260	1558080_s_at	5	*LOC144871*	300	215726_s_at	7	*CYB5A*
261	242352_at	5	*NIPBL*	301	207843_x_at	7	*CYB5A*
262	226184_at	5	*FMNL2*	302	209366_x_at	7	*CYB5A*
263	1565868_at	5	*CD44*	303	1558686_at	7	
264	242903_at	5	*IFNGR1*	304	212218_s_at	7	*FASN*
265	1557810_at	5		305	1555800_at	7	*ZNF385B*
266	225827_at	5	*EIF2C2*	306	243929_at	7	
267	205967_at	5	*HIST1H4C*	307	240024_at	7	*SEC14L2*
268	235425_at	5	*SGOL2*	308	225927_at	7	*MAP3K1*
269	211883_x_at	5	*CEACAM1*	309	1564786_at	7	*LOC338667*
270	221926_s_at	5	*IL17RC*	310	204607_at	7	*HMGCS2*
271	218182_s_at	5	*CLDN1*	311	206509_at	7	*PIP*
272	213446_s_at	5	*IQGAP1*	312	230238_at	7	*ANKRD43*
273	214336_s_at	5	*COPA*	313	239435_x_at	7	*SHROOM1*
274	220240_s_at	6	*TMCO3*	314	227702_at	7	*CYP4X1*
275	217777_s_at	6	*PTPLAD1*	315	223044_at	7	*SLC40A1*
276	224221_s_at	6	*VAV3*	316	233123_at	7	*SLC40A1*
277	208084_at	6	*ITGB6*	317	239723_at	7	
278	227314_at	6	*ITGA2*	318	214622_at	7	*CYP21A2*
279	213839_at	7	*KIAA0500*	319	235856_at	7	
280	235501_at	7		320	208451_s_at	7	*C4A / C4B*

Table S2-e List of 783 probe-sets used for classification of breast cancer

No.	Probeset ID	Gene cluster	Gene Symbol	No.	Probeset ID	Gene cluster	Gene Symbol
321	214428_x_at	7	*C4A / C4B*	361	202731_at	7	*PDCD4*
322	202357_s_at	7	*CFB*	362	212593_s_at	7	*PDCD4*
323	204070_at	7	*RARRES3*	363	212594_at	7	*PDCD4*
324	206396_at	7	*SLC1A1*	364	1553072_at	7	*BNIPL*
325	213664_at	7	*SLC1A1*	365	231084_at	7	*C10orf79*
326	211596_s_at	7	*LRIG1*	366	232381_s_at	7	*DNAH5*
327	205776_at	7	*FMO5*	367	238657_at	7	*UBXD3*
328	215300_s_at	7	*FMO5*	368	222068_s_at	7	*LRRC50*
329	223125_s_at	7	*C1orf21*	369	220173_at	7	*C14orf45*
330	218692_at	7	*GOLSYN*	370	241310_at	7	
331	208788_at	7	*ELOVL5*	371	225305_at	7	*SLC25A29*
332	225327_at	7	*KIAA1370*	372	232280_at	7	*SLC25A29*
333	212195_at	7	*IL6ST*	373	232290_at	7	
334	212196_at	7	*IL6ST*	374	226067_at	7	*C20orf114*
335	206869_at	7	*CHAD*	375	204378_at	7	*BCAS1*
336	226846_at	7	*PHYHD1*	376	207056_s_at	7	*SLC4A8*
337	223864_at	7	*ANKRD30A*	377	228175_at	7	
338	237339_at	7	*hCG_25653*	378	227177_at	7	*CORO2A*
339	228256_s_at	7	*EPB41L4A*	379	211695_x_at	7	*MUC1*
340	202962_at	7	*KIF13B*	380	207300_s_at	7	*F7*
341	204015_s_at	7	*DUSP4*	381	50965_at	7	*RAB26*
342	204014_at	7	*DUSP4*	382	234016_at	7	*ANKRD20B*
343	226034_at	7		383	240733_at	7	
344	228390_at	7		384	222804_x_at	7	*WDR32*
345	229072_at	7		385	230679_at	7	*WDR32*
346	201311_s_at	7	*SH3BGRL*	386	219001_s_at	7	*WDR32*
347	201312_s_at	7	*SH3BGRL*	387	226511_at	7	*WDR32*
348	239907_at	7		388	208473_s_at	7	*GP2*
349	205933_at	7	*SETBP1*	389	214324_at	7	*GP2*
350	213832_at	7		390	203355_s_at	7	*PSD3*
351	205453_at	7	*HOXB2*	391	204284_at	7	*PPP1R3C*
352	216603_at	7	*SLC7A8*	392	1558612_a_at	7	*ATP1A4*
353	202752_x_at	7	*SLC7A8*	393	227832_at	7	*MBD6*
354	216092_s_at	7	*SLC7A8*	394	203998_s_at	7	*SYT1*
355	1560550_at	7		395	213745_at	7	*ATRNL1*
356	217013_at	7	*LOC646282*	396	1563658_a_at	7	*SYT9*
357	203675_at	7	*NUCB2*	397	225911_at	7	*NPNT*
358	229838_at	7	*NUCB2*	398	214858_at	7	
359	1557867_s_at	7	*C9orf117*	399	218640_s_at	7	*PLEKHF2*
360	236085_at	7	*CAPSL*	400	222699_s_at	7	*PLEKHF2*

Table S2-f List of 783 probe-sets used for classification of breast cancer

No.	Probeset ID	Gene cluster	Gene Symbol	No.	Probeset ID	Gene cluster	Gene Symbol
401	203988_s_at	7	*FUT8*	441	205696_s_at	9	*GFRA1*
402	206346_at	7	*PRLR*	442	230163_at	9	*LOC143381*
403	206463_s_at	7	*DHRS2*	443	214440_at	9	*NAT1*
404	214079_at	7	*DHRS2*	444	227232_at	9	*EVL*
405	201349_at	7	*SLC9A3R1*	445	217838_s_at	9	*EVL*
406	243579_at	7	*MSI2*	446	244375_at	9	
407	201939_at	7	*PLK2*	447	225418_at	9	*PVRL2*
408	225915_at	7	*CAB39L*	448	1553622_a_at	9	*FSIP1*
409	205896_at	7	*SLC22A4*	449	212956_at	9	*TBC1D9*
410	227929_at	7		450	212960_at	9	*TBC1D9*
411	1556221_a_at	7		451	204798_at	9	*MYB*
412	239638_at	7		452	204667_at	9	*FOXA1*
413	240838_s_at	7	*LOC145837*	453	210085_s_at	9	*ANXA9*
414	225496_s_at	7	*SYTL2*	454	211712_s_at	9	*ANXA9*
415	232914_s_at	7	*SYTL2*	455	228241_at	9	*AGR3*
416	1560850_at	7		456	233388_at	9	
417	1567101_at	7		457	210735_s_at	9	*CA12*
418	205471_s_at	7	*DACH1*	458	215867_x_at	9	*CA12*
419	228915_at	7	*DACH1*	459	218195_at	9	*C6orf211*
420	219702_at	8	*PLAC1*	460	211235_s_at	9	*ESR1*
421	204531_s_at	8	*BRCA1*	461	205225_at	9	*ESR1*
422	206546_at	8	*SYCP2*	462	215551_at	9	*ESR1*
423	226446_at	8	*HES6*	463	232083_at	9	*C20orf23*
424	220898_at	8		464	241466_at	9	
425	1559949_at	8		465	214109_at	9	*LRBA*
426	244579_at	8		466	229381_at	9	*C1orf64*
427	224218_s_at	8	*TRPS1*	467	223103_at	9	*STARD10*
428	234351_x_at	8	*TRPS1*	468	232322_x_at	9	*STARD10*
429	221088_s_at	8	*PPP1R9A*	469	204623_at	9	*TFF3*
430	230290_at	8	*SCUBE3*	470	205009_at	9	*TFF1*
431	233273_at	9		471	223608_at	9	*EFCAB2*
432	242022_at	9		472	224443_at	9	*C1orf97*
433	219741_x_at	9	*ZNF552*	473	201825_s_at	9	*SCCPDH*
434	231820_x_at	9	*ZNF587*	474	201826_s_at	9	*SCCPDH*
435	223961_s_at	9	*CISH*	475	231018_at	9	*LOC342979*
436	238077_at	9	*KCTD6*	476	213201_s_at	9	*TNNT1*
437	235786_at	9		477	204540_at	9	*EEF1A2*
438	225064_at	9	*RABEP1*	478	221585_at	9	*CACNG4*
439	225092_at	9	*RABEP1*	479	210523_at	9	*BMPR1B*
440	227550_at	9	*LOC143381*	480	229975_at	9	

Table S2-g. List of 783 probe-sets used for classification of breast cancer

No.	Probeset ID	Gene cluster	Gene Symbol
481	204966_at	9	*BAI2*
482	243837_x_at	9	
483	209911_x_at	9	*HIST1H2BD*
484	230491_at	9	
485	205509_at	9	*CPB1*
486	207142_at	9	*KCNJ3*
487	233059_at	9	
488	210341_at	9	*MYT1*
489	217191_x_at	9	
490	230570_at	9	
491	216356_x_at	9	*BAIAP3*
492	227641_at	9	*FBXL16*
493	219051_x_at	9	*METRN*
494	232269_x_at	9	*METRN*
495	212686_at	9	*PPM1H*
496	205645_at	9	*REPS2*
497	227425_at	9	*REPS2*
498	236445_at	9	*LOC731986*
499	217133_x_at	9	*CYP2B6*
500	206754_s_at	9	*CYP2B7P1*
501	210272_at	9	*CYP2B7P1*
502	1562821_a_at	9	
503	237112_at	9	
504	1561938_at	9	
505	232034_at	9	*LOC203274*
506	211483_x_at	9	*CAMK2B*
507	215771_x_at	9	*RET*
508	223631_s_at	9	*C19orf33*
509	218657_at	9	*RAPGEFL1*
510	223658_at	9	*KCNK6*
511	242657_at	9	
512	207494_s_at	9	*ZNF76*
513	1552388_at	9	*FLJ30901*
514	212551_at	9	*CAP2*
515	235004_at	9	*RBM24*
516	231291_at	9	
517	209884_s_at	9	*SLC4A7*
518	206325_at	9	*SERPINA6*
519	233002_at	9	*KIAA1622*
520	226809_at	9	*FLJ30428*
521	242912_at	9	
522	1557843_at	9	
523	243241_at	9	
524	1563369_at	9	*FLJ42957*
525	219359_at	9	*ATHL1*
526	227379_at	9	*MBOAT1*
527	203639_s_at	9	*FGFR2*
528	208228_s_at	9	*FGFR2*
529	203638_s_at	9	*FGFR2*
530	207147_at	9	*DLX2*
531	235771_at	9	
532	1559739_at	9	*CHPT1*
533	221675_s_at	9	*CHPT1*
534	230364_at	9	*CHPT1*
535	205769_at	9	*SLC27A2*
536	218398_at	9	*MRPS30*
537	200648_s_at	9	*GLUL*
538	223551_at	9	*PKIB*
539	1553910_at	9	*NBPF4*
540	239337_at	9	*LOC400768*
541	241368_at	9	*LSDP5*
542	227966_s_at	9	*CCDC74A / 74B*
543	244745_at	9	*RERG*
544	209459_s_at	9	*ABAT*
545	209460_at	9	*ABAT*
546	227182_at	9	*SUSD3*
547	208305_at	9	*PGR*
548	228554_at	9	
549	209339_at	9	*SIAH2*
550	243907_at	9	
551	205440_s_at	9	*NPY1R*
552	1563512_at	9	*NOS1AP*
553	215153_at	9	*NOS1AP*
554	218094_s_at	9	*SYS1-DBNDD2*
555	1567997_x_at	9	
556	1556551_s_at	9	*SLC39A6*
557	231713_s_at	9	*ELP2*
558	240633_at	9	*DOK7*
559	203685_at	9	*BCL2*
560	232210_at	9	

Table S2-h List of 783 probe-sets used for classification of breast cancer

No.	Probeset ID	Gene cluster	Gene Symbol	No.	Probeset ID	Gene cluster	Gene Symbol
561	232614_at	9		601	206091_at	9	*MATN3*
562	201860_s_at	9	*PLAT*	602	231518_at	9	*LOC283867*
563	205948_at	9	*PTPRT*	603	222379_at	9	*KCNE4*
564	219197_s_at	9	*SCUBE2*	604	223235_s_at	9	*SMOC2*
565	229764_at	9	*FAM79B*	605	201667_at	9	*GJA1*
566	209681_at	9	*SLC19A2*	606	217764_s_at	9	*RAB31*
567	242662_at	9	*PCSK6*	607	226736_at	9	*CHURC1*
568	204045_at	9	*TCEAL1*	608	231859_at	9	*C14orf132*
569	243967_at	9	*AFF3*	609	225363_at	9	*PTEN*
570	241577_at	9		610	205239_at	9	*AREG*
571	244696_at	9		611	206115_at	9	*EGR3*
572	216381_x_at	9	*AKR7A3*	612	208606_s_at	9	*WNT4*
573	205355_at	9	*ACADSB*	613	231944_at	9	*ERO1LB*
574	226030_at	9	*ACADSB*	614	218541_s_at	9	*C8orf4*
575	237350_at	9	*LOC143941*	615	202458_at	9	*PRSS23*
576	1555893_at	9		616	204663_at	9	*ME3*
577	212637_s_at	9	*WWP1*	617	206143_at	9	*SLC26A3*
578	202554_s_at	9	*GSTM3*	618	212741_at	9	*MAOA*
579	209443_at	9	*SERPINA5*	619	227722_at	9	*RPS23*
580	229158_at	9	*WNK4*	620	236222_at	9	*C3orf15*
581	1560260_at	9	*LOC285593*	621	228528_at	9	
582	219438_at	9	*NKAIN1*	622	232603_at	9	*DCDC5*
583	220540_at	9	*KCNK15*	623	233413_at	9	
584	212492_s_at	9	*JMJD2B*	624	227742_at	9	*CLIC6*
585	212495_at	9	*JMJD2B*	625	242913_at	9	*CLIC6*
586	212496_s_at	9	*JMJD2B*	626	207144_s_at	9	*CITED1*
587	1563367_at	9		627	205710_at	9	*LRP2*
588	203928_x_at	9	*MAPT*	628	230863_at	9	*LRP2*
589	206401_s_at	9	*MAPT*	629	232850_at	9	
590	203929_s_at	9	*MAPT*	630	1554007_at	9	
591	225379_at	9	*MAPT*	631	228905_at	9	*PCM1*
592	224909_s_at	9	*PREX1*	632	201963_at	10	*ACSL1*
593	224925_at	9	*PREX1*	633	225984_at	10	*PRKAA1*
594	235049_at	9	*ADCY1*	634	217276_x_at	10	*SERHL2*
595	205380_at	9	*PDZK1*	635	217284_x_at	10	*SERHL*
596	243168_at	9		636	1553410_a_at	10	*ABCC12*
597	223600_s_at	9	*KIAA1683*	637	224146_s_at	10	*ABCC11*
598	236114_at	9		638	204942_s_at	10	*ALDH3B2*
599	210374_x_at	9	*PTGER3*	639	211237_s_at	10	*FGFR4*
600	213933_at	9	*PTGER3*	640	1553394_a_at	10	*TFAP2B*

Table S2-i. List of 783 probe-sets used for classification of breast cancer

No.	Probeset ID	Gene cluster	Gene Symbol	No.	Probeset ID	Gene cluster	Gene Symbol
641	214451_at	10	*TFAP2B*	681	219087_at	12	*ASPN*
642	219580_s_at	10	*TMC5*	682	224396_s_at	12	*ASPN*
643	222904_s_at	10	*TMC5*	683	238478_at	12	*BNC2*
644	1553434_at	10	*CYP4Z2P*	684	213290_at	12	*COL6A2*
645	237395_at	10	*CYP4Z1*	685	212489_at	12	*COL5A1*
646	227971_at	10	*NRK*	686	213125_at	12	*OLFML2B*
647	231098_at	10		687	229084_at	12	*CNTN4*
648	204351_at	11	*S100P*	688	202410_x_at	12	*IGF2 / INS-IGF2*
649	201884_at	11	*CEACAM5*	689	204345_at	12	*COL16A1*
650	203757_s_at	11	*CEACAM6*	690	206101_at	12	*ECM2*
651	211657_at	11	*CEACAM6*	691	209550_at	12	*NDN*
652	1553436_at	11	*MUC19*	692	205381_at	12	*LRRC17*
653	226960_at	11	*CXCL17*	693	214761_at	12	*ZNF423*
654	241031_at	11	*FAM148A*	694	204519_s_at	13	*PLLP*
655	224576_at	11	*ERGIC1*	695	227762_at	13	
656	230906_at	11	*GALNT10*	696	244697_at	13	
657	234785_at	11		697	210051_at	13	*RAPGEF3*
658	210130_s_at	11	*TM7SF2*	698	225987_at	13	*STEAP4*
659	219300_s_at	11	*CNTNAP2*	699	230661_at	13	*LOC286191*
660	219301_s_at	11	*CNTNAP2*	700	213900_at	13	*C9orf61*
661	206378_at	11	*SCGB2A2*	701	204731_at	13	*TGFBR3*
662	206799_at	11	*SCGB1D2*	702	226625_at	13	*TGFBR3*
663	220622_at	11	*LRRC31*	703	222862_s_at	13	*AK5*
664	214774_x_at	11	*TOX3*	704	228653_at	13	*SAMD5*
665	226455_at	11	*CREB3L4*	705	240724_at	13	
666	236256_at	11		706	204636_at	13	*COL17A1*
667	215559_at	11	*ABCC6*	707	216918_s_at	13	*DST*
668	214307_at	11	*HGD*	708	238827_at	13	
669	205221_at	11	*HGD*	709	225728_at	13	*SORBS2*
670	214308_s_at	11	*HGD*	710	236656_s_at	13	
671	209959_at	12	*NR4A3*	711	225123_at	13	
672	242836_at	12		712	223168_at	13	*RHOU*
673	243296_at	12	*PBEF1*	713	208798_x_at	13	*GOLGA8A*
674	213931_at	12	*ID2 /// ID2B*	714	236307_at	13	
675	227952_at	12	*ZNF718*	715	226147_s_at	13	*PIGR*
676	215440_s_at	12	*BEX4*	716	229659_s_at	13	
677	204412_s_at	12	*NEFH*	717	201525_at	13	*APOD*
678	227194_at	12	*FAM3B*	718	215271_at	13	*TNN*
679	206994_at	12	*CST4*	719	232570_s_at	13	*ADAM33*
680	237411_at	12	*ADAMTS6*	720	207761_s_at	13	*METTL7A*

Table S2-j. List of 783 probe-sets used for classification of breast cancer

No.	Probeset ID	Gene cluster	Gene Symbol
721	244655_at	13	*LOC644192*
722	242868_at	13	
723	1555564_a_at	13	*CFI*
724	212558_at	13	*SPRY1*
725	230130_at	13	
726	228750_at	13	
727	209540_at	13	*IGF1*
728	209541_at	13	*IGF1*
729	214920_at	13	*THSD7A*
730	243481_at	13	*RHOJ*
731	227874_at	13	*EMCN*
732	231947_at	13	*MYCT1*
733	200795_at	13	*SPARCL1*
734	232935_at	13	
735	202920_at	13	*ANK2*
736	209763_at	13	*CHRDL1*
737	202242_at	13	*TSPAN7*
738	207542_s_at	13	*AQP1*
739	209047_at	13	*AQP1*
740	227725_at	13	*ST6GALNAC1*
741	208891_at	13	*DUSP6*
742	208892_s_at	13	*DUSP6*
743	1553602_at	13	*MUCL1*
744	206164_at	13	*CLCA2*
745	206165_s_at	13	*CLCA2*
746	217528_at	13	*CLCA2*
747	218899_s_at	13	*BAALC*
748	202948_at	13	*IL1R1*
749	203305_at	13	*F13A1*
750	213139_at	13	*SNAI2*
751	205582_s_at	13	*GGTLA1*
752	212358_at	13	*CLIP3*
753	205542_at	13	*STEAP1*
754	221841_s_at	13	*KLF4*
755	201041_s_at	13	*DUSP1*
756	227404_s_at	13	*EGR1*
757	204803_s_at	13	*RRAD*
758	207317_s_at	13	*CASQ2*
759	213006_at	13	*CEBPD*
721	244655_at	13	*LOC644192*
760	1560049_at	13	
761	203088_at	13	*FBLN5*
762	222043_at	13	*CLU*
763	209686_at	13	*S100B*
764	229839_at	13	*SCARA5*
765	235849_at	13	*SCARA5*
766	203435_s_at	13	*MME*
767	238018_at	13	*FAM150B*
768	209576_at	13	*GNAI1*
769	203324_s_at	13	*CAV2*
770	206953_s_at	13	*LPHN2*
771	208370_s_at	13	*RCAN1*
772	212724_at	13	*RND3*
773	215933_s_at	13	*HHEX*
774	226931_at	13	*TMTC1*
775	211026_s_at	13	*MGLL*
776	228766_at	13	*CD36*
777	241929_at	13	
778	205913_at	13	*PLIN*
779	207175_at	13	*ADIPOQ*
780	201785_at	13	*RNASE1*
781	202723_s_at	13	*FOXO1*
782	221268_s_at	13	*SGPP1*
783	222773_s_at	13	*GALNT12*

Table S3 Thirty probe-sets representing cell-cycle and proliferation genes were used to show distinctive differential expression of cell-cycle and proliferation genes among different molecular subtypes of breast cancer. These genes were identified as part of a common neoplastic signature from our previous study of paired hepatocelluar and adjacent non-tumorous liver tissues.

Affymetrix Probe-set ID	Gene Symbol
201292_at	*TOP2A*
202580_x_at	*FOXM1*
202705_at	*CCNB2*
202715_at	*CAD*
202870_s_at	*CDC20*
203109_at	*UBE2M*
203213_at	*CDC2*
203554_x_at	*PTTG1*
204641_at	*NEK2*
204720_s_at	*DNAJC6*
204768_s_at	*FEN1*
204822_at	*TTK*
204825_at	*MELK*
205047_s_at	*ASNS*
205393_s_at	*CHEK1*
207165_at	*HMMR*
207828_s_at	*CENPF*
209035_at	*MDK*
209408_at	*KIF2C*
209464_at	*AURKB*
209714_s_at	*CDKN3*
215090_x_at	*NPEPPS*
217714_x_at	*STMN1*
218009_s_at	*PRC1*
218355_at	*KIF4A*
218663_at	*HCAP-G*
219494_at	*RAD54B*
219918_s_at	*ASPM*
219978_s_at	*NUSAP1*
219990_at	*E2F8*

Table S4 Probe-set IDs and genes from the OncotypeDX and MammaPrint predictors that were used to score risk of distant recurrence. Sixteen genes in the OncotypeDX predictor can be matched to Affymetrix probe-set IDs and NKI-ID. Forty eight out of seventy MammaPrint predictor genes can be matched to Affymetrix probe-set IDs in the U133A GeneChip and used for the study.

OncotypeDX Predictor Genes

Gene Symbol	Affymetrix Probeset ID	NKI ID
BAG1	202387_at	ID5227
CD68/EIF4A1	203507_at	ID22119
BCL2	203685_at	ID22945
ESR1	205225_at	ID18904
PGR	208305_at	ID630
SCUBE2	219197_s_at	ID10658
GSTM1	204550_x_at	ID22320
GRB7	210761_s_at	ID7930
ERBB2	216836_s_at	ID6424
CTSL2	210074_at	ID22839
MMP11	203878_s_at	ID13284
CCNB1	214710_s_at	ID14976
MKI67	212023_s_at	ID1161
MYBL2	201710_at	ID1354
AURKA	208079_s_at	ID5281
BIRC5	202094_at	ID21371

MammaPrint Predictor Genes

Gene Symbol	Affymetrix Probeset ID	NKI ID
AKAP2	202759_s_at	ID12009
ALDH4	211552_s_at	ID6556
AP2B1	200612_s_at	ID22282
BBC3	211692_s_at	ID12695
CCNE2	205034_at	ID8994
CEGP1	219197_s_at	ID10658
CENPA	204962_s_at	ID1944
COL4A2	211964_at	ID2146
DC13	218447_at	ID3476
DCK	203302_at	ID23739
DHX58	219364_at	ID18440
DIAPH3	220997_s_at	ID22739
ECT2	219787_s_at	ID23213
ESM1	208394_x_at	ID10260
EXT1	201995_at	ID18906
FGF18	211029_x_at	ID7474
FLJ11190	219958_at	ID19709
FLT1	204406_at	ID22706
GMPS	214431_at	ID7504
GNAZ	204993_at	ID22879
GSTM3	202554_s_at	ID24348
HEC	204162_at	ID8746
HSA250839	219686_at	ID20335
IGFBP5	211959_at	ID22447
IGFBP5	211959_at	ID12587
KIAA0175	204825_at	ID14112
KIAA1067	212248_at	ID16531
L2DTL	218585_s_at	ID16238
LOC51203	218039_at	ID15405
LOC57110	219983_at	ID5373
MCM6	201930_at	ID13145
MMP9	203936_s_at	ID10842
MP1	205273_s_at	ID14907
NMU	206023_at	ID13324
ORC6L	219105_x_at	ID10243
OXCT	202780_at	ID21365
PECI	218025_s_at	ID8797
PECI	218025_s_at	ID9171
PK428	203794_at	ID5308
PRC1	218009_s_at	ID8523
RAB6B	210127_at	ID16966
RFC4	204023_at	ID5529
SERF1A	219982_s_at	ID20881
SLC2A3	202499_s_at	ID15609
TGFB3	209747_at	ID1846
TSPYL5	213122_at	ID10904
UCH37	219960_s_at	ID17793
WISP1	206796_at	ID7524

Table S5. Survival comparison between patients treated with CMF and CAF adjuvant chemotherapy in each molecular subtype of breast cancer.

Breast cancer subtype	Patient No.		p value of Log-rank test (CAF vs. CMF)	
	CAF	CMF	Metastasis.-free survival	Overall survival
I	10	13	0.82	0.82
II	5	6	0.62	0.76
III	16	4	0.58	0.51
IV	22	17	7.00E-05	0.002
V	12	8	0.41	0.96
VI	22	11	0.23	0.06

Table S6 Statistical comparison of pertinent clinical parameters between subtype I patients treated with CAF and CMF adjuvant chemotherapy.

		CAF<br>n= 10		CMF<br>n= 13	Fisher exact test<br>p value
**Age at diagnosis**					1
< 50 yr	7	70.0%	9	69.2%	
>= 50 yr	3	30.0%	4	30.8%	
**TNM Path T**					0.38
1	2	20.0%	6	46.2%	
2	8	80.0%	7	53.8%	
**TNM Path N**					0.17
0	5	50.0%	11	84.6%	
1	5	50.0%	2	15.4%	
**TNM Path M**					
0	10	100.0%	13	100.0%	
**Positive Lymph Nodes**					0.17
0	5	50.0%	11	84.6%	
1-3	5	50.0%	2	15.4%	
**TNM Stage**					0.09
I	1	10.0%	6	46.2%	
II	9	90.0%	7	53.8%	
**Nuclear Grade**					
1	0	0.0%	1	7.7%	0.49
2	1	10.0%	2	15.4%	
3	9	90.0%	9	69.2%	
**Hormonal Therapy**					0.62
No	7	70.0%	11	84.6%	
Yes	3	30.0%	2	15.4%	
**Post-op Radiation**					0.65
No	6	60.0%	10	76.9%	
Yes	4	40.0%	3	23.1%	
