# Supplementary material for: Correlation of microarray-based breast cancer molecular subtypes and clinical outcomes: implications for treatment optimization
Source: BMC Cancer. 2011 Apr 18;11:143. doi: 10.1186/1471-2407-11-143 (PMC3094326; doi:10.1186/1471-2407-11-143)
Supplement: Additional file 2 — Supplemental Methodology. Methodology includes four sections: I) procedures for selection of classification probe-sets and molecular subtyping by two steps k-means clustering analysis; II) determination of cut-point values for estrogen receptor (ER), progesterone receptor (PR) and HER2; III) scoring relative risk of distant recurrence using genes of the OncotypeDX and MammaPrint predictor; and IV) statistical comparison for concordance of differential gene expression patterns among six breast cancer subtypes between KFSYSCC dataset and public datasets from EMC (ref. [10]), Uppsala (ref. [19]), and TRANSBIG (ref. [20]). [file 1471-2407-11-143-S2.PDF]

Additional file 2

Supplemental Methodology

for

Correlation of Microarray-based Breast Cancer Molecular Subtypes and Clinical Outcomes: Implications for Treatment Optimization

Kuo-Jang Kao, Kai-Ming Chang, Hui-Chi Hsu and Andrew T. Huang

I. Procedures for selection of classification probe-sets and molecular subtyping by two steps k-means clustering analysis
II. Determination of cut-point values for estrogen receptor (ER), progesterone receptor (PR) and HER2.
III. Scoring relative risk of distant recurrence using the genes of OncotypeDX and MammaPrint predictor.
IV. Statistical comparison for concordance of differential gene expression patterns among six breast cancer subtypes between KFSYSCC dataset and public datasets from EMC, Uppsala, and TRANSBIG.

Methodology

I. Procedures for Selection of Classification Probe-sets and for Molecular Subtyping of Breast Cancer by Two Steps k-means Clustering Analysis

Selection of Probe-Sets for Classification of Breast Cancer Molecular Subtypes:

To define breast cancer molecular subtype according to gene expression profiling, we took the following five steps to select appropriate probe sets for classification.

Step 1. Genes that have been reported to play important roles in human breast cancer in the literature were identified as pivotal genes (n=23) (Additional file 1, Table S1).

Step 2. An Affymetrix probe-set was chosen to represent each pivotal gene. If there were more than one probe-set for a pivotal gene, a representing probe-set was chosen according to the following two criteria: i) a probe-set should express higher intensity and a wider range among 312 samples; and ii) the same probe-set should show good linear correlation with most of other probe-sets representing the same gene.

Step 3. A linear and a quadratic correlation were conducted between the representing probe-set of each pivotal gene and all other probe-sets on the U133 Plus 2.0 array in all 312 samples of the cohort 1. Probe-sets showing good proportional or reverse linear ($p<10^{-10}$) or nonlinear quadratic correlation ($p <10^{-5}$) with the probe set of each pivotal gene were identified and selected.

Step 4. The identified probe-sets were further selected according to the following four criteria: **i)** normalized expression intensities of a selected probe-set must be >512 in at least 5 out of a total of 312 arrays; **ii)** fold change of normalized expression intensities between the samples at 10% quantile and 90% quantile must be > 4; **iii)** kurtosis of distribution of normalized expression intensities for a probe set in all 312 samples has to be smaller than zero **(**Determination of kurtosis is detailed in the later section.); **iv)** number of peaks on the first derivative of the density function of 312 samples should be greater than 1 (Determination of peak is detailed in the later section.). These four criteria were used to identify highly robust probes-sets with potential to differentiate different subtypes of breast cancer. We identified 1,144 probe-sets that met our criteria.

Step 5. Our earlier unpublished study revealed that differential expression of immune response genes play a significant role in determining the survival outcome within the same subtypes of breast cancer. Immune response likely varies between different individuals within the same molecular subtypes. Inclusion of immune response genes for subtyping could further split a major molecular subtype and complicate classification. For this reason, we identified immune response genes by identification of those probe-sets with their expression linearly or quadratically correlated with the expression intensities of CD19 (a major marker for B lymphocytes) (Affymetrix probe set ID 206398_s_at) and CD3D (a major marker for T lymphocytes) (Affymetrix probe set ID 213539_at). These genes are likely associated with B-cell or T-cell immune responses, and were excluded from the 1,144 selected probe sets.

After exclusion of the immune response genes, a total of 768 probe sets were obtained. The 768 probe-sets included 8 probe-sets from the 23 pivotal genes that passed the intensity filters (Step 4). The remaining 15 pivotal genes that didn't meet the intensity filter of "step 4" were added back to the 768 genes. The final number of total probe-sets available for classification of breast cancer was 783 (Supplements: Table S2).

Kurtosis and Peak:

Kurtosis measures how peaked or flat the data are relative to a normal distribution. Here we define kurtosis as $\frac{\frac{1}{n}\sum_{i=1}^{n}(X_i - \overline{X})^2}{s^4} - 3$, where $s^2 = \frac{1}{n-1}\sum_{i=1}^{n}(X_i - \overline{X})^2$. Data with small kurtosis indicated data had a flatter distribution and heavily tailed, and data with large kurtosis indicated they had a sharper peak and lightly tailed. The kurtosis of a normal distribution under this definition is 0. We therefore selected genes with kurtosis < 0, because they have broader distribution.

The density curve of gene expression among samples was approximated using the density function (default setting) in R statistical package from Bioconductor (http://www.bioconductor.org). The curve was smoothed by a Gaussian kernel.

We define peaks as the local maxima if a data curve (x_i, y_i), i=1,…, p.

First, we define a window width 2k+1, where 1≤(2k+1)≤p; (x_j, y_j) is a peak if y_j is the maximum amongst y_{j-k}, y_{j-k+1}, …, y_{j+k-1}, y_{j+k} for all k<i<(p-k), and x_j is the location of the peak. In practice, if there are several maxima within a window, we consider the maximum at left as the local maximum. The local maximum of within a window is a peak only when it locates at the middle of the window. In our case, we set k=25. We use this criteria to pick genes with distributions that have more than one peak.

Clustering Analysis for Identification of Breast Cancer Molecular Subtypes:

For the study, we first ran a hierachical clustering analysis using the 783 probe sets on all 327 samples in the cohorts 1 and 2. The result indicates that there were potentially 6 or 8 different major subtypes of breast cancer. We then conducted k means clustering analyses using a 2-step method. The 2-step method was implemented by using built-in default "*kmeans*" and "*hclust*" function in the R software package (v2.6) from Bioconductor (http://www.bioconductor.org). Average linkage and (1-Pearson correlation coefficient) as distance matrix were set for k means clustering analysis. The 2-step method was conducted as following:

Step 1- We ran k means clustering in R software for a given k of 8. After a k means clustering analysis, an integer cluster label from 1 to 8 can be assigned to each breast cancer sample. The cluster analysis was repeated 2000 times using random initial group center assigned by R package. Consequently each sample had a secondary sets of data consisted of 2000 k-means cluster labels as integer numbers from 1 to 8 for each sample.

Step 2 - Three hundred and twenty seven breast cancer samples were analyed by hierarchical clustering using 2,000 cluster labels of each sample. The hierarchical clustering procedure of 2,000 cluster labels on 327 breast cancer samples was performed using the average-linkage and a distance metric defined by 1-r, where r is the Pearson correlation between samples. The samples clusters can be determined by setting a uniform height of 1-r shown in the figure below. The samples connected by any sub-dendrogram set at height of 1-r were mutually correlated with correlation of r. By changing the cut point 1-r, we could vary the number of groups of the samples. Three

examples of setting “r” at 0.04 (red), 0.07 (green) and 0.2 (blue) were shown in the figure below. When 1-r was set at 0.96, there were 6 different sample clusters.

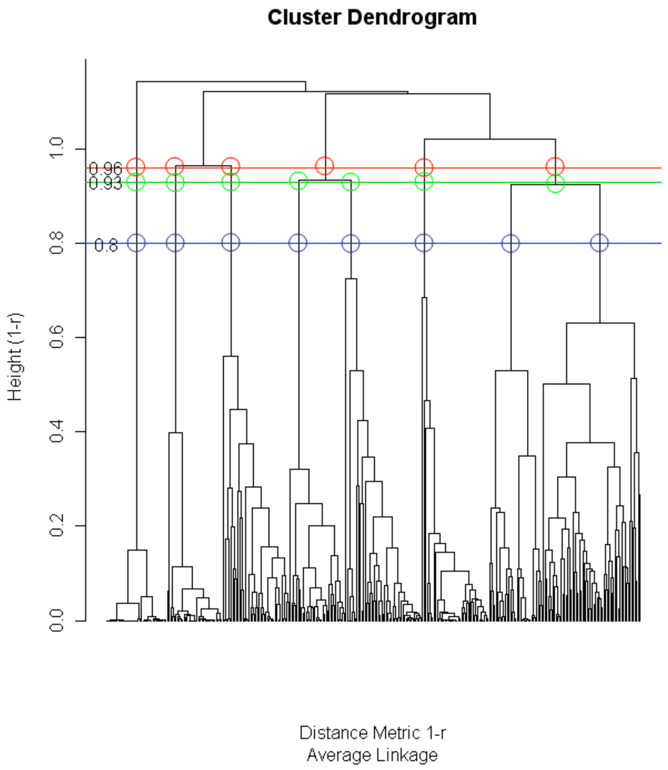

We then applied the method proposed by Smolkin and Ghosh (BMC Bioinformatics 4:36-42, 2003) to assess stability of sample clusters determined at different Pearson correlation values.

The first assessment was performed as following:

Eighty percent of 327 samples were randomly sampled twice to generate a pair of sub-datasets. The 2000 cluster labels generated for each sample by k-means clustering analyses as described earlier were used to conduct hierachical clustering analysis for each pair of sub-datasets, separately. The samples were clustered into different numbers of groups (e.g. g=2, 3, 4 …..,11) according to different Pearson correlation values as described above. The similarity between results of each pair for each number of groups (g=2, 3, 4….,11) was measured by calculation of Jaccard coefficient (JC). The closer the JC is to 1, the more similar two separate clustering results are. This process was repeated

200 times. The hitograms of 200 sets of JCs for each number of groups (g=2 to 11) are shown in the following figures.

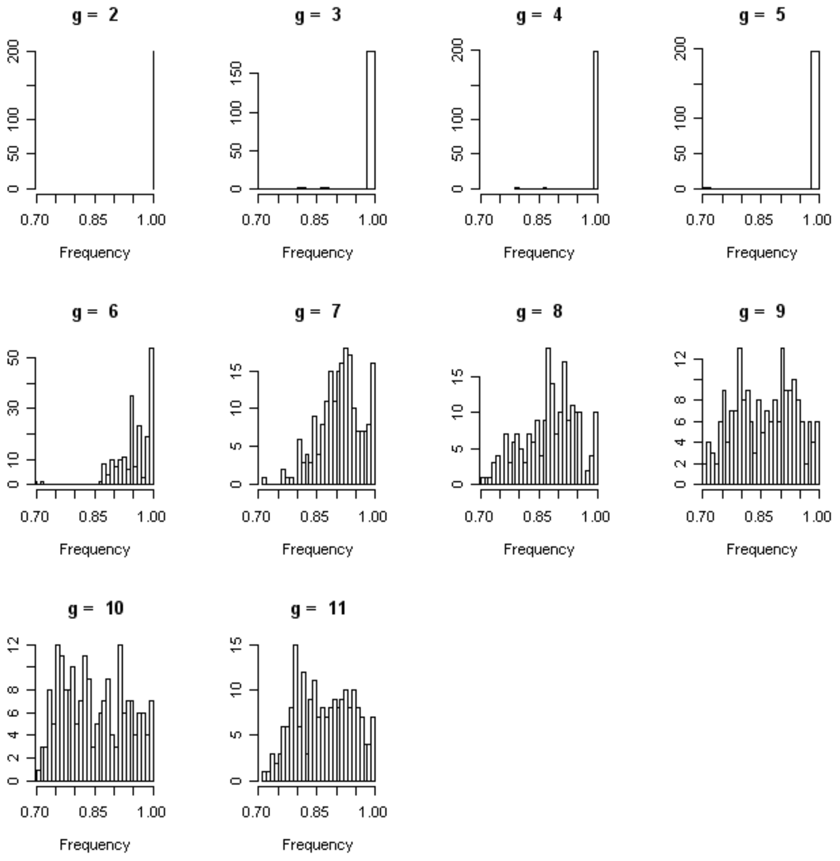

Figure legend: The histograms of the Jaccard coefficients given different number of clusters based on 200 paired random sub-sampled hierarchical cluster analyses. The number of clusters is stable when the Jaccard coefficients were closed to 1.

The second assessment was also conducted to determine average stability of different number of breast cancer groups generated at different height (1-r). For this assessment, a hierarchical clustering analysis was conducted using 2000 k-means cluster labels for each sample to create a full dendrogram of 327 samples. Samples were clustered into different number of groups by cutting the dendrogram at different height levels (1-r).

Next, a hierarchical clustering analysis was conducted using 80% of the 2000 k-means cluster labels which were randomly selected for each sample to create a dendrogram of 327 samples. Samples were clustered into different number of groups at

different heights (1-r). This clustering analysis was repeated 200 times. The percentage for cases remain in the same group by the full dendrogram was calculated as a stability measurement of the groups

The average of stability measurements for each cluster (sample group) was taken as the average group stability score reflecting how unlikely the group was due to chance.

The stability scores of each group for different number of groups from 4 to 11 were shown in following table.

k=8	Group 1	Group 2	Group 3	Group 4	Group 5	Group 6	Group 7	Group 8	Group 9	Group 10	Group 11	Average Stability
4 Groups	81	134	37	75								
Group Stability	92.5	71.5	100	96.5								90.1
5 Groups	81	93	37	75	41							
Group Stability	92.5	98.5	100	96.5	72							91.9
6 Groups	81	93	37	34	41	41						
Group Stability	92	98	100	100	96.5	72						93.1
7 Groups	47	93	37	34	41	34	41					
Group Stability	75.5	64	100	100	65	66	72					77.5
8 Groups	47	33	37	34	60	41	34	41				
Group Stability	58.5	100	100	100	98.5	96.5	100	72				90.7
9 Groups	46	33	37	34	60	41	34	41	1			
Group Stability	64.5	97	97	97	95.5	96.5	97	26	45			79.5
10 Groups	46	33	37	34	60	41	34	40	1	1		
Group Stability	67.5	98	98	96.5	59	95.5	98	98	59	59		82.9
11 Groups	46	33	37	34	53	41	34	40	7	1	1	
Group Stability	59	95.5	95.5	94	95.5	67	95.5	95.5	86	92.5	69	85.9

Based on the results from the method proposed by Smolkin and Ghosh (BMC Bioinformatics 4:36-42, 2003), we chose groups of 6 for our breast cancer molecular subtypes.

II. Determination of Cut-point Values for Positivity of Estrogen Receptor (ER), Progesterone Receptor (PR) and HER2

For determination of gene expression cut-point values that can be used to decide whether a breast cancer sample is positive or negative for ER, PR or HER2, we plotted density plot of all 312 samples from cohort 1 (Additional file 2: Fig. S1). The results showed bimodal distributions (negative vs. positive). We then applied the following statistical method to determine the cut-point values (C).

Suppose x is the observed expression of a marker for a sample. The posterior probabilities of the case being from the negative population and the positive populations are denoted as $P(-|x)$ and $P(+|x)$, respectively. Let $D(x)=P(+|x)/P(-|x)$, the decision function is:

$$\delta(x)=\begin{cases}\text{positive status} & \text{if } \dfrac{P(+\mid x)}{P(-\mid x)}>\text{d or } D(x)>\text{d}\\ \text{negative status} & \text{Otherwise}\end{cases},$$

Where d is a constant. In our case, we set **d** to be 1. That is, if the probability of the case being in the positive population is greater than the probability of the case of being in the negative population, than the case is said to be of positive status; otherwise, the case is said to be of negative status.

According to the Bayes rule,

$P(\mathrm{k}|x) = \pi_k P(x|\mathrm{k}) / p(x)$

where k is either + or -, and $P(x|\mathrm{k})$ is the probability of x being observed (if the case is truly from population k), π_k is the prior probability of the case being from population k ($\pi_{k+}+\pi_{k-}=1$), and $p(x)$ is the marginal probability of observing x.

As a result,

$$D(x)=\frac{\pi_+ P(x\mid +)}{\pi_- P(x\mid -)}.$$

We assume x follows a normal distribution with mean μ_k and variance σ_k^2, where k is either + or -. A cut-point **C** can be derived so that the decision function is equivalent to:

$$\delta(x)=\begin{cases}\text{positive status} & \text{if } x>\text{C}\\ \text{negative status} & \text{Otherwise}\end{cases}$$

That is, if x is smaller than the cut-point, the case is then decided to be from the negative population; otherwise, the case is from the positive population. The prior probability π_- is reparameterized as $1/[1+\exp(-t)]$ for computational purpose.

We have $\mathrm{C}=\dfrac{-b-\sqrt{b^2-4ac}}{2a}$ if a>0 and $\mathrm{C}=\dfrac{-b+\sqrt{b^2-4ac}}{2a}$ if a<0 if a<0

where

$$a=\sigma_-^2-\sigma_+^2,$$

$$b=2\times(\mu_-\sigma_+^2-\mu_+\sigma_-^2),$$

$$c=\sigma_-^2\mu_+^2-\sigma_+^2\mu_-^2-2\sigma_-^2\sigma_+^2\left[-t+\ln(\frac{\sigma_-}{\sigma_+})\right].$$

In our case, μ_{-}, μ_{+} , σ_{-}^{2}, σ_{k+}^{2}, and *t* are unknown and are estimated by their maximum likelihood estimators (MLEs). The MLEs of μ_{-} , μ_{+}, σ_{-}^{2}, σ_{k+}^{2}, and *t* were derived using the default non-linear minimization (**nlm**) function (Newton-type method) in R package software (v2.6.0) based on 312 cases in the cohort 1. Initial point for the **nlm** function was subjectively selected to ensure a reasonable solution.

We were interesting in ER, PR and HER2 status. They were represented by the probe-sets 205225_at, 208305_at and 216836_s_at, respectively.

The cut-point and the estimation for the parameters were:

	cut-point	m-	s-	m+	s+	t
ER	11.61956	9.3574	1.4737	13.3138	0.8059	-0.4281
Her2	13.26387	11.2639	0.8321	14.432	0.569	1.1612
PR	4.141207	2.9724	0.6992	7.3942	1.6947	-1.3304

Initial points for fitting the MLEs for the parameters

	$\mu-$	$\sigma-$	$\mu+$	$\sigma+$	τ
ER	8	1	14	1	-1
Her2	8	1	14	1	1
PR	2	1	10	1	1

The cut-point values to determine statuses of ER, PR and HER2 as listed above were 11.6, 4.1 and 13.3, respectively (Figure S1). The values were logarithm of normalized expression intensity to a base of 2.

III. Scoring of Relative Risk for Distant Recurrence Using the OncotypeDX and MammaPrint Predictors.

We applied the predictive models of van't Veer *et al.* (ref. 3) (MammaPrint) and Paik *et al.* (ref. 7) (OncotypeDX) to our dataset and the datasets of EMC (ref. 9) and NKI (ref. 23) to determine the relative risk for distant recurrence. To calculate the recurrence score of Oncotype DX, the model of Paik *et al.* (ref. 8) involving 16 genes associated

with distant recurrence was directly applied all three datasets. Probe-sets of Affymetrix U133A GeneChip and genes of NKI DNA microarray corresponding to the 16 genes were identified (Additional file 1, Table S4). Expression intensities of these 16 genes were fed into the model directly to calculate the recurrence score of each case. For the NKI dataset, quantile-normalized red channel data were used to determine gene expression intensities. To calculate the score correlated with low risk of distant recurrence using the genes of MammaPrint predictor (ref. 3), we identified 48 Affymetrix probe-sets matched to the Mammaprint predictor (Additional file 1, Table S4). We then determined the Pearson correlation coefficient of each sample with the average good prognosis profile of the NKI dataset. The average good prognosis profile was established by calculation of the average gene expression intensity of the 44 low-risk cases reported in the study of van't Veer et al. (ref. 3) for each gene used in the predictor.

IV. Statistical comparison for concordance of differential gene expression patterns between KFSYSCC dataset and public datasets from EMC (ref. 10), Uppsala (ref. 19), and TRANSBIG (ref. 20).

The primary purpose of this study is to determine the concordance of differential gene expression pattern of four signatures associated with cell cycle/proliferation (A), wound response (B), stromal reaction (C), and tumor vascular endothelial normalization (D) among six breast cancer molecular subtypes between our cohort and each of the three published independent cohorts (refs. 10, 19, 20). For each cohort, we used genes in each signature to draw a heat map according to the results of one-way hierachical clustering analysis (Additional File 3, Figure S4). The concordance of the heat map patterns between KFSYSCC cohort and each of Uppsala (19), EMC (10), and TRANSBIG (20) cohorts was statistically measured and tested as described below.

Method

The gene expression data were quantile-normalized. Z score of each gene for each sample was calculated in each cohort. Next, we determined the average of Z scores for each molecular subtype in each cohort. The average Z scores were used to draw a heat map for each signature and cohort. The heat map was drawn according to the dendrogram of genes in each signature as shown in Additional file 3, Figure S4 for each cohort.

The concordance of gene expression pattern at the molecular subtype level for each gene signature between 2 cohorts was determined by Pearson correlation. The significance of each correlation coefficient was tested by comparing the correlation coefficient to the empirical null distribution of the correlation coefficients derived from 10,000 permutations of molecular subtypes at sample level.

Results

The heat maps of average Z scores for each gene and molecular subtype were shown in Figures A-D below. Figures A-D demonstrated similar expression patterns at molecular subtype level among four different cohorts. The levels of concordance between KFSYSCC cohort and other cohorts for four different gene signatures were analyzed by Pearson correlation. The results summarized in Table A below showed high degree of concordance between our cohort and each of the other three independent cohorts. The p values for all coefficients are highly significant ($p<10^{-4}$). These results validate the molecular subtypes determined with our classification genes.

Figures A-D. Heat maps of average Z-score of each molecular subtype in each cohort for each signature are shown below.

Figure A. Cell Cycle/Proliferation

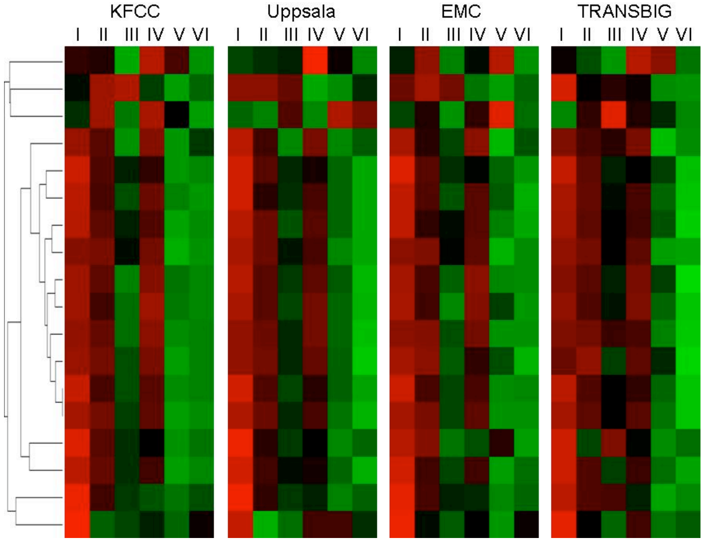

Figure B. Wound Response

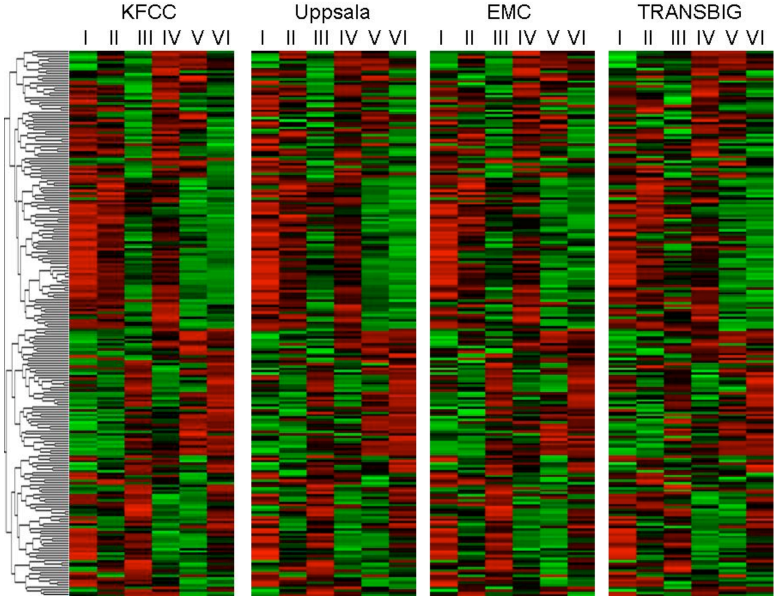

Figure C. Stromal Reaction

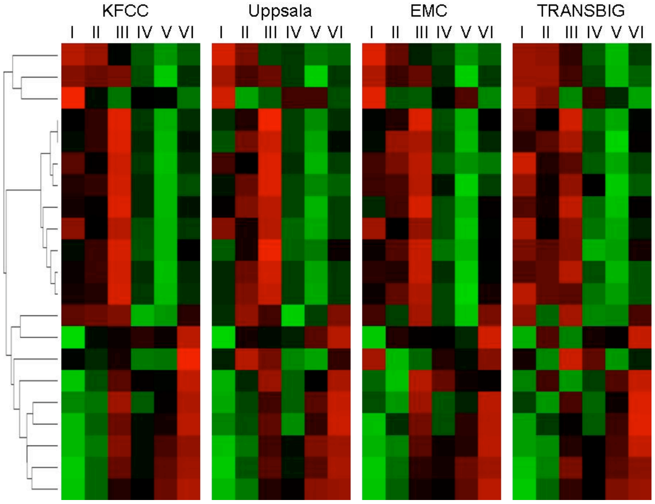

Figure D. Tumor vascular endothelial normalization

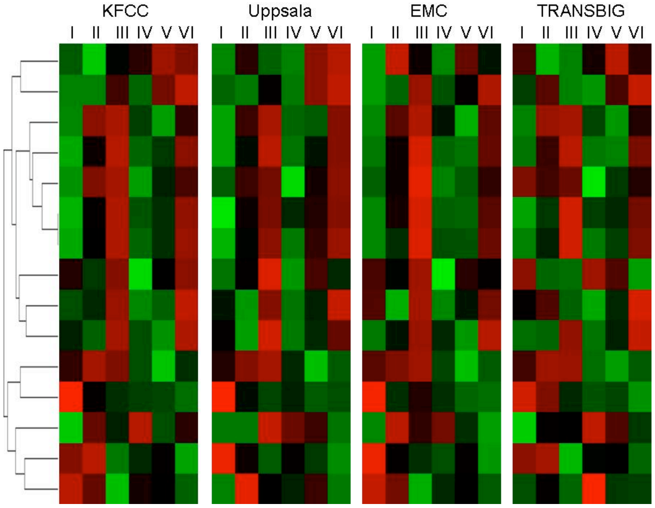

Table A. Pearson correlation coefficients for each signature between the KFSYSCC cohort and each of the other three cohorts (EMC, Uppsala and TRANSBIG). P-values for all correlation coefficients are $<10^{-4}$.

Signature	Uppsala	EMC	TRANSBIG
**Cell Cycle/Proliferation**	0.92	0.94	0.87
**Wound Response**	0.84	0.85	0.78
**Stromal Reaction**	0.91	0.94	0.87
**Vascular Normalization**	0.86	0.86	0.83
