# Supplementary material for: Correlation of microarray-based breast cancer molecular subtypes and clinical outcomes: implications for treatment optimization
Source: BMC Cancer. 2011 Apr 18;11:143. doi: 10.1186/1471-2407-11-143 (PMC3094326; doi:10.1186/1471-2407-11-143)

Additional File 3

Supplemental Figures

for

Correlation of Microarray-based Breast Cancer Molecular Subtypes and Clinical Outcomes: Implications for Treatment Optimization

Kuo-Jang Kao, Kai-Ming Chang, Hui-Chi Hsu and Andrew T. Huang

Figures S1-S7

Figure S1 Cut-points to determine positivity of ER, PR and HER2.

Figure S2 Correlation studies between immunohistochemistry and gene expression results for ER, PR and HER2 statuses.

Figure S3 Functional annotation of gene clusters for breast cancer molecular subtyping.

Figure S4 Dendrograms of genes associated with cell cycle/proliferation, stromal reaction, wound response and vascular endothelial normalization for characterization of breast cancer molecular subtypes.

Figure S5 Differential expression of the selected genes by breast cancer molecular subtypes in different datasets.

Figure S6 Comparison of metastasis-free survival between subtypes V and VI breast cancer patients classified as Perou-Sørlie luminal A intrinsic type in patients of the present study.

Figure S7 Differential expression of genes associated with epithelial-mesenchymal transition among breast cancer molecular subtypes of the present study.

Figure S1 Density plots of estrogen receptor (ER), progesterone receptor (PR) and HER2 using 312 breast cancer samples in the cohort 1 to determine the cut-points for positivity and negativity. The cut-points are shown by intercepts with green lines. Y-axis represents relative number of samples and X-axis represents logarithm of expression intensity to base 2 for ER, PR or HER2. The cut-point values for ER, PR and HER2 were 11.6, 4.1 and 13.2, respectively. The method is detailed in methodology of the additional files.

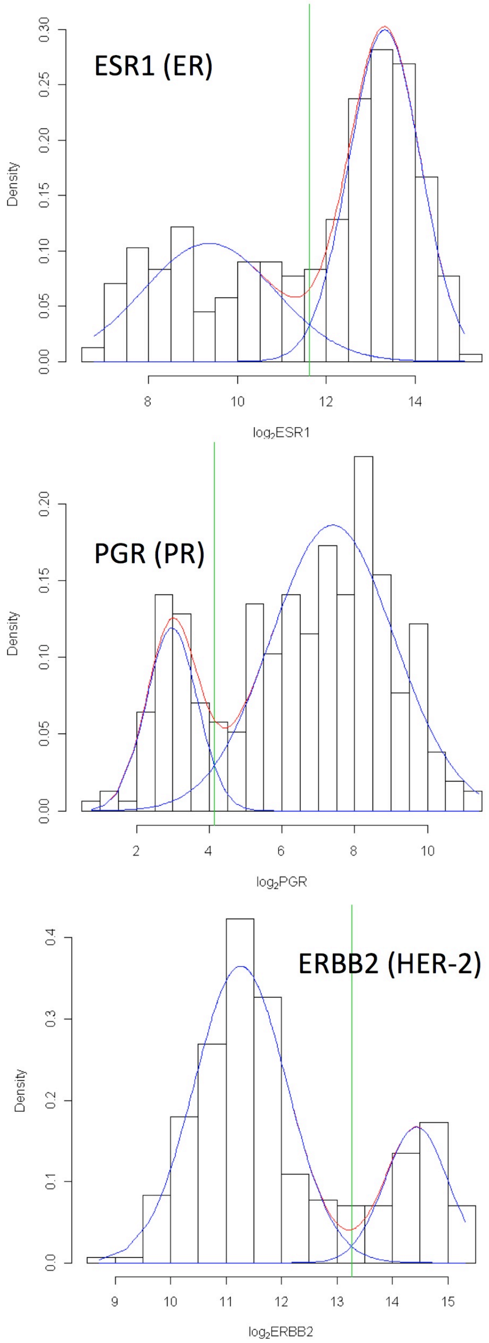

Figure S2. Correlation studies between immunohistochemistry and gene expression results for ER, PR and HER2 statuses. The cut-point for determination of positivity and negativity of ER, PR or HER2 was indicated by red dash lines. Numbers of cases above and below the cut-points are shown in each panel. Analyses by Kappa statistics showed significant degree of concordance between Microarray and IHC results.

a) Correlation study for ER

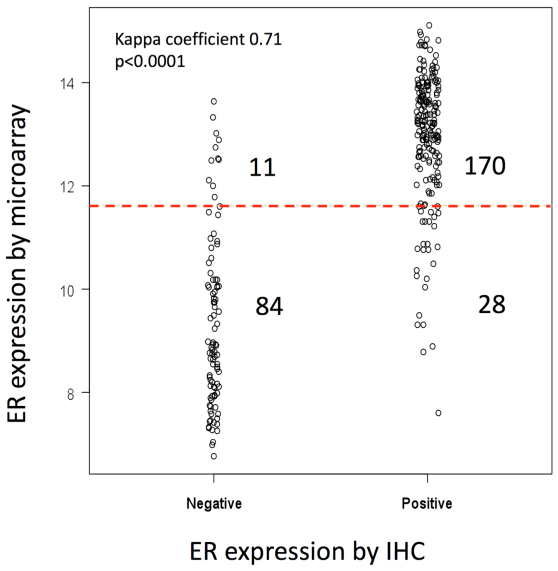

b) Correlation study for HER2

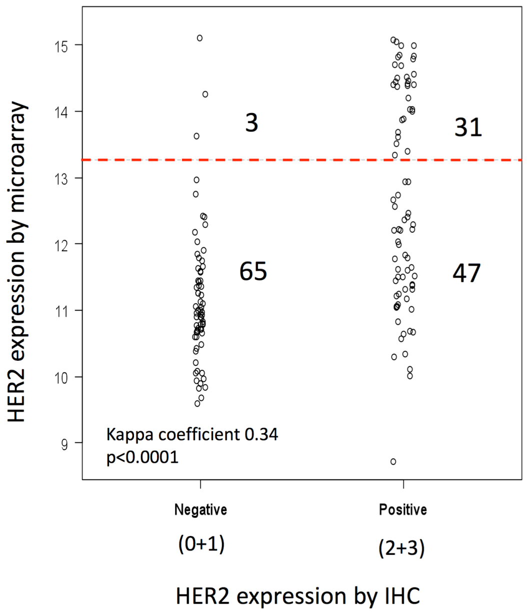

c) Correlation for PR

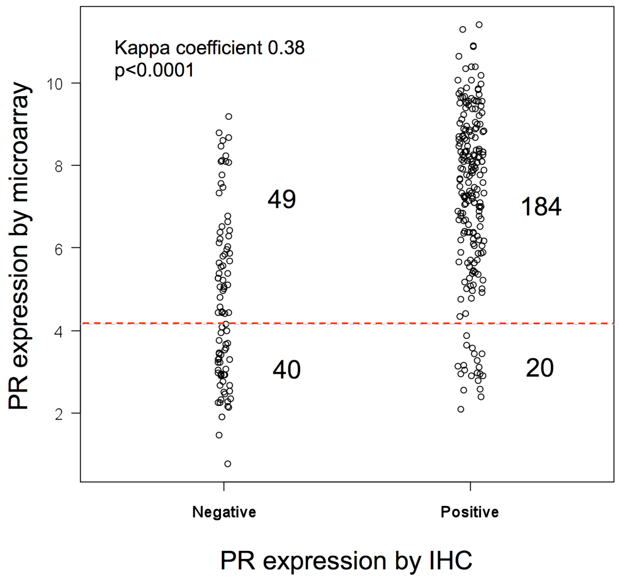

Fig. S3 Functional annotation of 13 gene clusters generated by hierachical clustering analysis using 783 probe sets on 327 breast cancer samples. Dendrogram of all 783 probe-sets is shown at left. The 13 gene clusters were determined by visual inspection of the dendrogram. Genes in each cluster are listed in Table S2, and were analyzed for enrichment of certain molecular and cellular functions using Ingenuity Pathway Analysis (https://analysis.ingenuity.com/pa/). The values in parentheses were –log (p value) for enrichment of each molecular and cellular function.

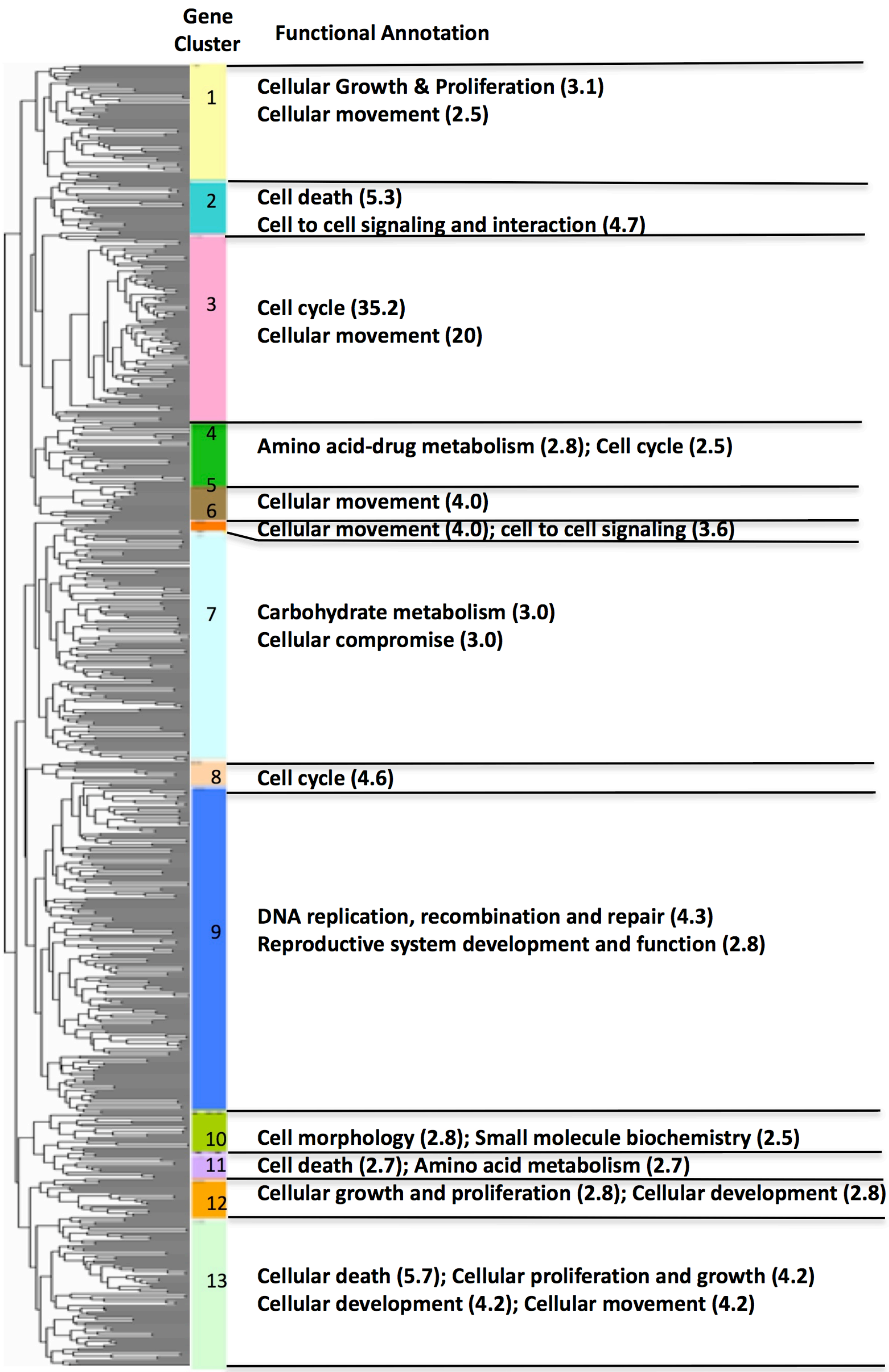

Figure S4. Genes/probe-sets of different signatures used to characterize molecular subtypes of breast cancer.

C. Wound Response Signature Genes

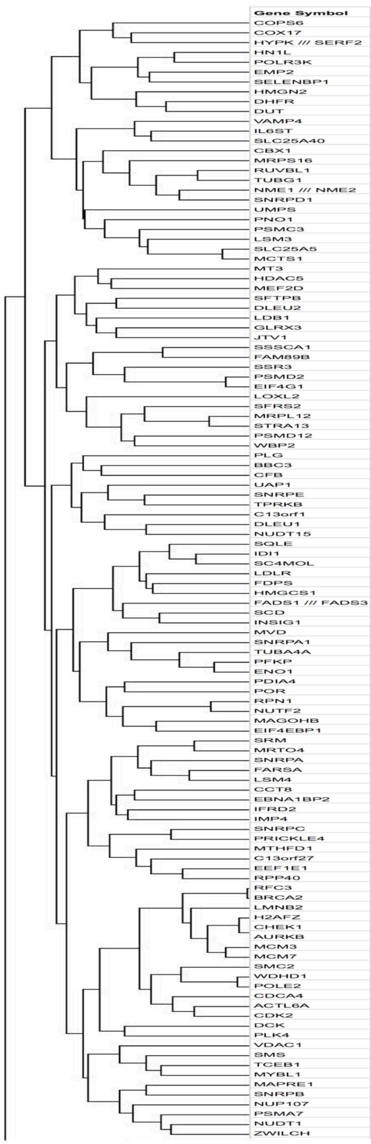

Gene Symbol	Affy. Probeset
COPS6	213504_at
COX17	203880_at
HYPK /// SERF2	217756_x_at
HN1L	212109_at
POLR3K	218866_s_at
EMP2	204975_at
SELENBP1	214433_s_at
HMGN2	208668_x_at
DHFR	202534_x_at
DUT	208955_at
VAMP4	211760_s_at
IL6ST	204864_s_at
SLC25A40	205716_at
CBX1	201518_at
MRPS16	218046_s_at
RUVBL1	201614_s_at
TUBG1	201714_at
NME1 /// NME2	201577_at
SNRPD1	202691_at
UMPS	202707_at
PNO1	211697_x_at
PSMC3	201267_s_at
LSM3	202209_at
SLC25A5	200657_at
MCTS1	218163_at
MT3	205970_at
HDAC5	202455_at
MEF2D	203003_at
SFTPB	213936_x_at
DLEU2	216870_x_at
LDB1	35160_at
GLRX3	207506_at
JTV1	209972_s_at
SSSCA1	203114_at
FAM89B	212484_at
SSR3	217790_s_at
PSMD2	200830_at
EIF4G1	208625_s_at
LOXL2	202999_s_at
SFRS2	200754_x_at
MRPL12	203931_s_at
STRA13	209478_at
PSMD12	202353_s_at
WBP2	209117_at
PLG	209977_at
BBC3	211692_s_at
CFB	211920_at
UAP1	209340_at
SNRPE	203316_s_at
TPRKB	219030_at
C13orf1	214668_at
DLEU1	205677_s_at
NUDT15	219347_at
SQLE	209218_at
IDI1	208881_x_at
SC4MOL	209146_at
LDLR	202068_s_at
FDPS	201275_at
HMGCS1	221750_at
FADS1 /// FADS3	208964_s_at
SCD	200832_s_at
INSIG1	201626_at
MVD	203027_s_at
SNRPA1	206055_s_at
TUBA4A	212242_at
PFKP	201037_at
ENO1	201231_s_at
PDIA4	208658_at
POR	208928_at
RPN1	201011_at
NUTF2	202397_at
MAGOHB	218894_s_at
EIF4EBP1	221539_at
SRM	201516_at
MRTO4	220688_s_at
SNRPA	201770_at
FARSA	202159_at
LSM4	202737_s_at
CCT8	200873_s_at
EBNA1BP2	201323_at
IFRD2	209100_at
IMP4	212411_at
SNRPC	201342_at
PRICKLE4	218233_s_at
MTHFD1	202309_at
C13orf27	213346_at
EEF1E1	204905_s_at
RPP40	213427_at
RFC3	204127_at
BRCA2	214727_at
LMNB2	216952_s_at
H2AFZ	213911_s_at
CHEK1	205394_at
AURKB	209464_at
MCM3	201555_at
MCM7	208795_s_at
SMC2	213253_at
WDHD1	204727_at
POLE2	205909_at
CDCA4	218399_s_at
ACTL6A	202666_s_at
CDK2	204252_at
DCK	203302_at
PLK4	211088_s_at
VDAC1	212038_s_at
SMS	202043_s_at
TCEB1	202823_at
MYBL1	213906_at
MAPRE1	200713_s_at
SNRPB	208821_at
NUP107	218768_at
PSMA7	201114_x_at
NUDT1	204766_s_at
ZWILCH	218349_s_at

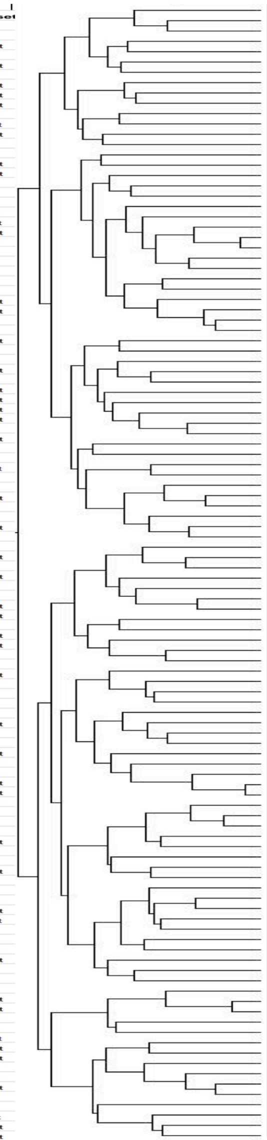

PNN	212037_at
PLXNB1	215807_s_at
ZNF219	219314_s_at
IGBP1	202105_at
MYCBP	203361_s_at
PDK2	202590_s_at
MNAT1	203565_s_at
MFSD11	221192_x_at
ZBTB17	203602_s_at
KIAA0090	212394_at
SLC25A36	201919_at
LPA /// PLG	209978_s_at
RAB3B	205924_at
CTNS	36566_at
F10	205620_at
MBP	209072_at
GATM	203178_at
BCL6	203140_at
ARHGEF3	218501_at
REV3L	208070_s_at
KIT	205051_s_at
CDKN1C	213348_at
GSN	200696_s_at
DPYSL2	200762_at
ADD3	201034_at
ADAMTS1	222162_s_at
PKIG	202732_at
BDH2	218285_s_at
F3	204363_at
OLFML2A	213075_at
GNG11	204115_at
JAG1	216268_s_at
KIAA0323	212356_at
KIAA1305	220911_s_at
CNTNAP1	219400_at
GABBR1	203146_s_at
SLC22A17	218675_at
PLA2R1	210194_at
CLCN6	203950_s_at
BHLHB2	201170_s_at
CALCOCO1	209002_s_at
KIAA1109	212779_at
GPLD1	206264_at
PBXIP1	212259_s_at
MXI1	202364_at
RAB11FIP2	203883_s_at
TNFSF12	205611_at
CST3	201360_at
FCGRT	218831_s_at
CRTAP	201380_at
CAMLG	203538_at
CTSF	203657_s_at
HAS2	206432_at
PLOD2	202620_s_at
ENTPD4 /// LOXL	202998_s_at
MYL6 /// MYL6B	212082_s_at
FLNC	207876_s_at
LUM	201744_s_at
DNM1	215116_s_at
LSS	211018_at
PTPRU	211320_s_at
SLC5A3	212944_at
SDC1	201286_at
TNFRSF12A	218368_s_at
MAP3K8	205027_s_at
CDKN1A	202284_s_at
JUNB	201473_at
ID3	207826_s_at
MET	213807_x_at
RNF146	221430_s_at
WTAP	203137_at
PNRC1	209034_at
ID2	201566_x_at
IL1R1	215561_s_at
MAF	206363_at
SERPING1	200986_at
DAB2	201278_at
ACTG2	202274_at
TAGLN	205547_s_at
MFGE8	210605_s_at
SVIL	202565_s_at
TPM1	210986_s_at
TPM2	212654_at
APP	200602_at
GLS	203159_at
DUSP22	218845_at
MSN	200600_at
LPIN1	212276_at
SATB1	203408_s_at
C10orf10	209183_s_at
ITGA6	201656_at
EPHB1	210753_s_at
SLC16A1	202235_at
ITPKB	203723_at
CYP27A1	203979_at
PIK3IP1	221756_at
IL7R	205798_at
CLIC2	213415_at
STK17A	202694_at
NCOA3	209061_at
TNFAIP2	202510_s_at
CSF1	209716_at
CKLF	221058_s_at
CORO1C	221676_s_at
TIMP2	203167_at
PLAUR	214866_at
FABP3	214285_at
LIPA	201847_at
PLD3	201050_at
C2	203052_at

D. Tumor Vascular Endothelial Normalization Signature Genes

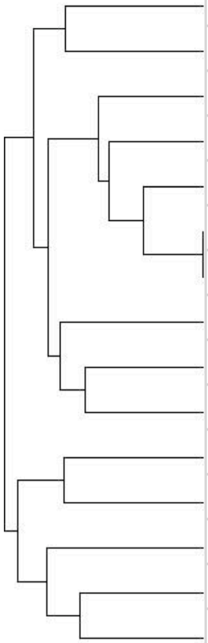

Gene Symbol	Affy. Probeset ID
VEGFB	203683_s_at
PDGFC	218718_at
VEGFC	209946_at
EPAS1	200878_at
KDR	203934_at
TIE1	204468_s_at
CDH5	204677_at
FLT4	210316_at
PDGFA	205463_s_at
FIGF	206742_at
HIF1A	200989_at
EGLN1	221497_x_at
PDGFB	204200_s_at
VEGFA	210513_s_at
FLT1	210287_s_at

Figure S4. Genes/probe-sets of different signatures used to characterize molecular subtypes of breast cancer.

A. Cell Cycle/Proliferaion Signature Genes

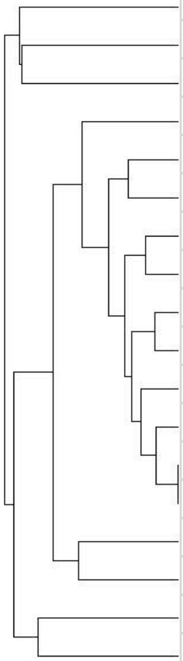

Gene Symbol	Affy. Probeset ID
UBE2M	203109_at
MDK	209035_at
NPEPPS	215090_x_at
RAD54B	219494_at
CHEK1	205393_s_at
FEN1	204768_s_at
AURKB	209464_at
E2F8	219990_at
NEK2	204641_at
TOP2A	201291_s_at
CDC2	203213_at
NUSAP1	219978_s_at
FOXM1	202580_x_at
HCAP-G	218663_at
CAD	202715_at
STMN1	217714_x_at
ASNS	205047_s_at
DNAJC6	204720_s_at

B. Stromal Reaction Signature Genes

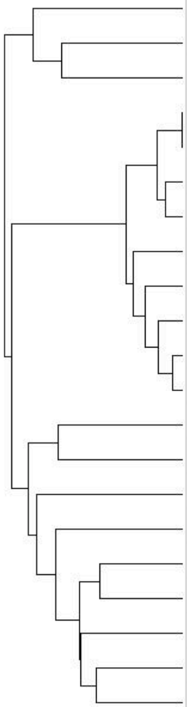

Gene Symbol	Affy. Probeset ID
HRASLS	219983_at
ADM	202912_at
SPP1	209875_s_at
CD48	204118_at
CD52	204661_at
LCP1	208885_at
PLEK	203471_s_at
CD8A	205758_at
RUNX3	204198_s_at
GIMAP5	218805_at
CD247	210031_at
GZMA	205488_at
HOXA10	213150_at
ITGBL1	205422_s_at
SNTG2	220487_at
F2RL2	206795_at
FRZB	203697_at
OGN	218730_s_at
CXCL14	218002_s_at
ADRA2A	209869_at
RAI2	219440_at

Figure S5 A-E. Scatter and box plots of gene expression by different breast cancer molecular subtypes in four independent datasets. The five genes used in this study were chosen for their roles in drug sensitivity and epithelial-mesenchymal transition of breast cancer cells. None of them were part of the genes used for classification of molecular subtypes. As shown in these figures, all four different datasets shared the same differential distribution patterns according to the six molecular subtypes. The expression intensities of these genes among six molecular subtypes were significantly different according to ANOVA except ZEB1 in the EMC dataset. The Y-axis is logarithm of gene expression intensity to base 2. The four datasets are ours (KFSYSCC), TRANSBIG [20], EMC [9] and Uppsala [19].

Fig. S5-A. CAV1 gene. P values of ANOVA test for KFSYSCC, TRANSBIG, EMC, and Uppsala datasets are $9.3x10^{-35}$, $2.7x10^{-9}$, $4.9x10^{-21}$ and $2.9x10^{-30}$, respectively.

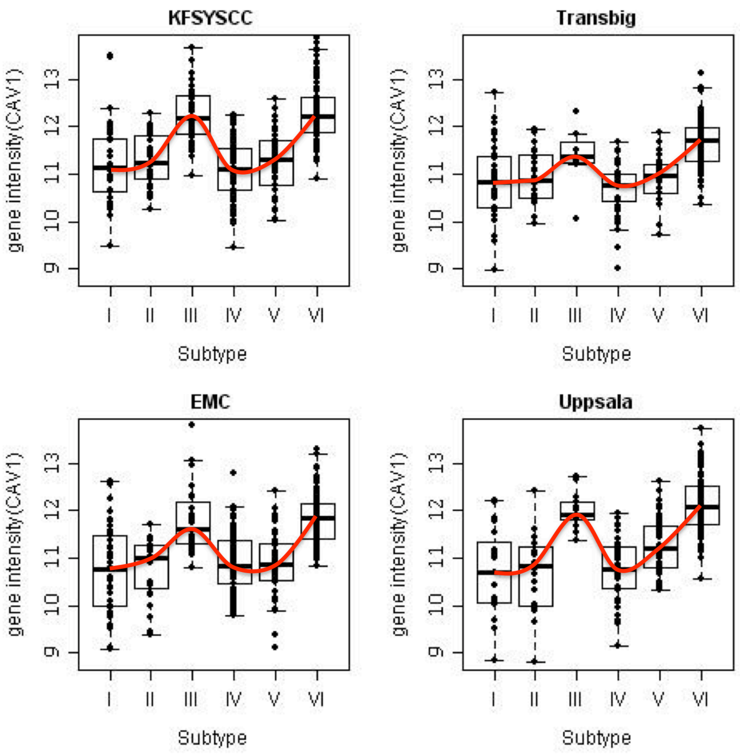

Fig. S5-B. DHFR Gene. P values of ANOVA test for KFSYSCC, TRANSBIG, EMC and Uppsala datasets are 8.6x10-[14], $8.3x10^{-6}$, $3.3x10^{-4}$ and $2.8x10^{-11}$, respectively.

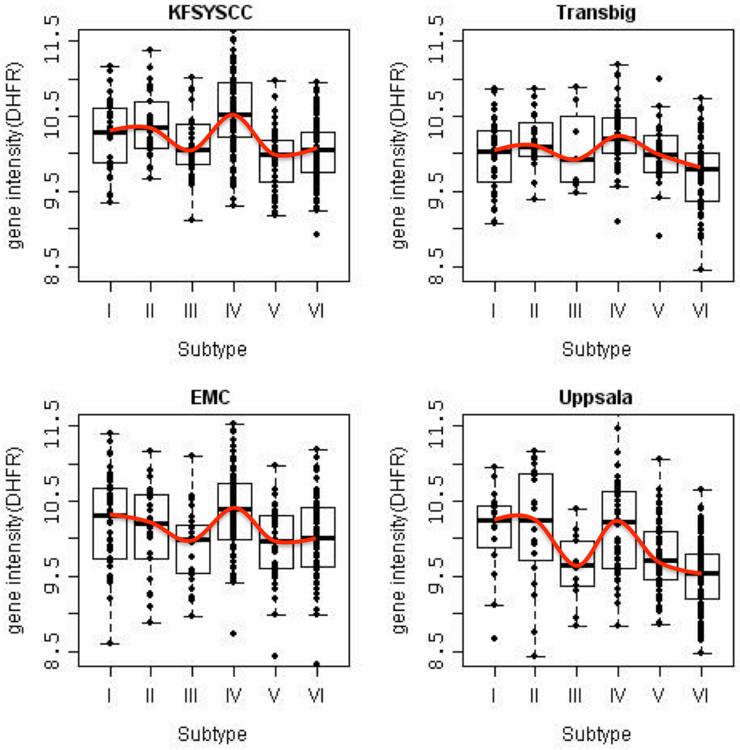

Fig. S5-C. TYMS gene. P values of ANOVA test for KFSYSCC, TRANSBIG, EMC and Uppsala datasets are $8.4x10^{-36}$, $1.5x10^{-23}$, $5.0x10^{-29}$ and $9.8x10^{-30}$, respectively.

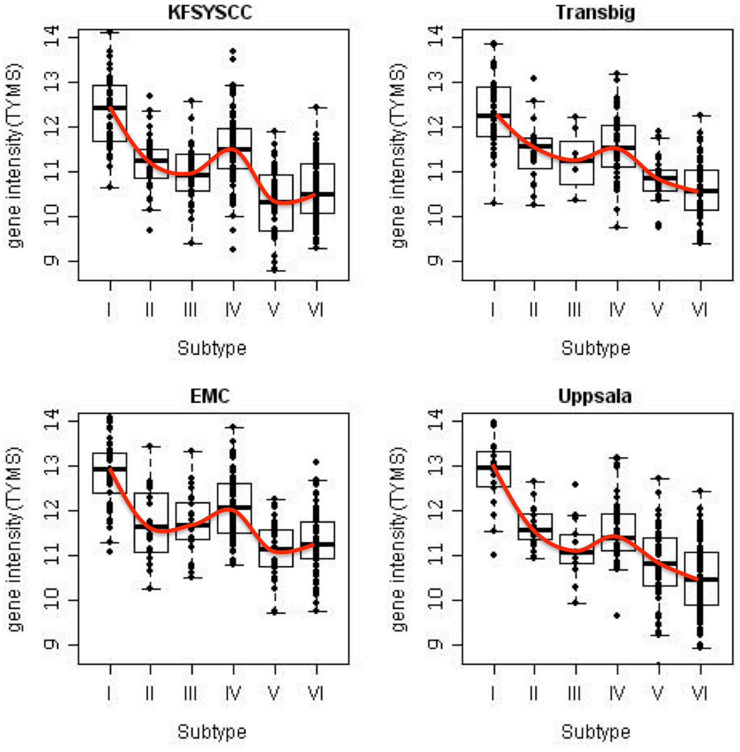

Fig. S5-D. VIM gene. P values of ANOVA test for KFSYSCC, TRANSBIG, EMC, and Uppsala datasets are 1.8x10-^{17}, 1.3x10^{-8}, 4.7x10^{-15} and 3.1x10^{-16}, respectively.

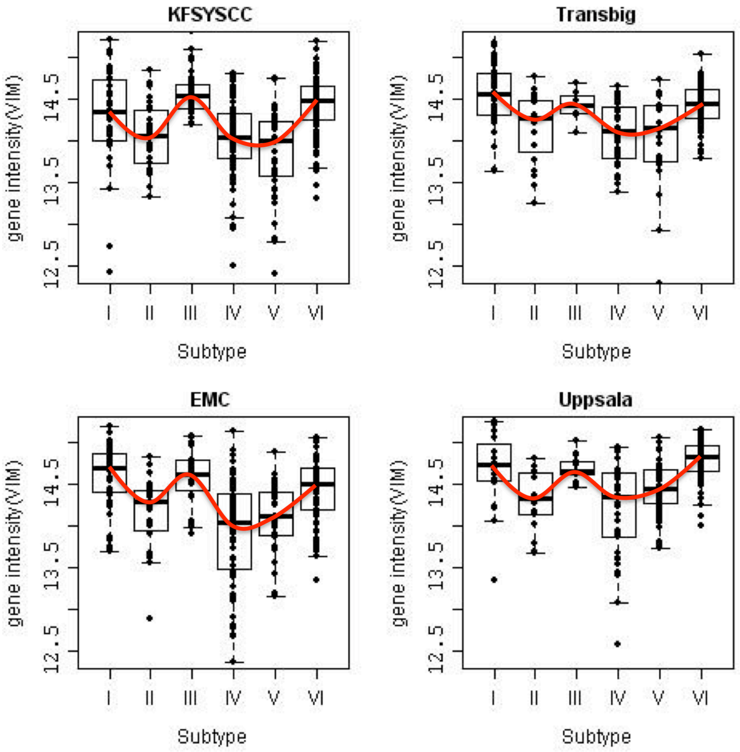

Fig. S5-E. ZEB1 gene. P values of ANOVA test for KFSYSCC, TRANSBIG, EMC and Uppsala datasets are 2.1x10^{-16}, 0.05, 0.07 and 6.7x10^{-7}, respectively.

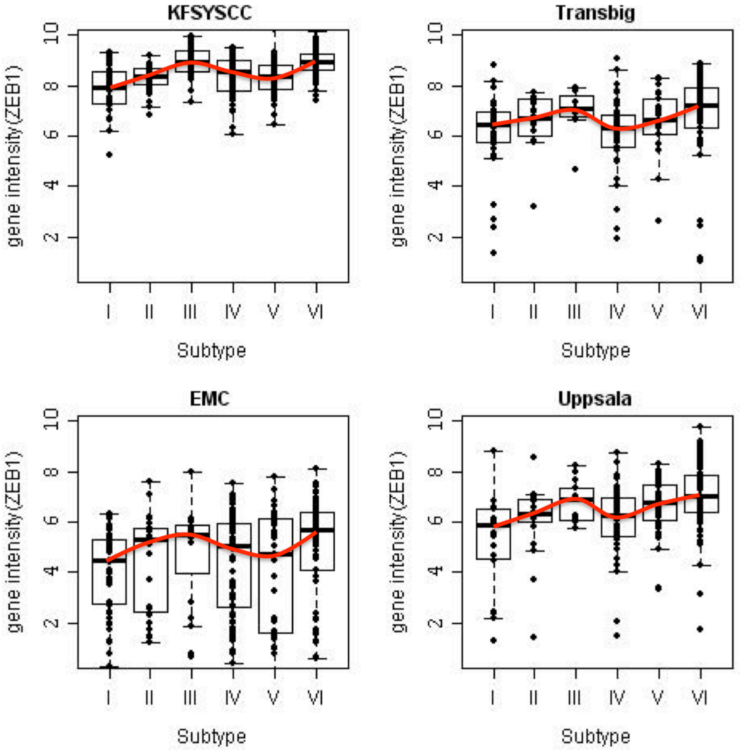

Figure S6 Comparison of metastasis-free survival between subtypes V and VI breast cancer patients classified as Perou-Sørlie luminal A intrinsic type in patients of the present study.

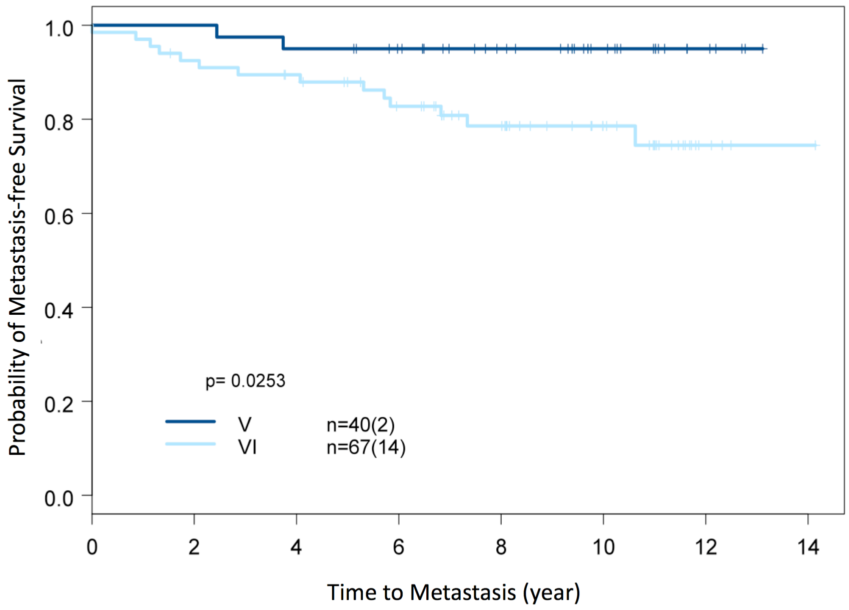

Figure S7 Differential expression of genes associated with epithelial-mesenchymal transition among breast cancer molecular subtypes of the present study. The solid colored dots and bars represent mean ±SD. P values were determined by ANOVA. Expression of each gene is logarithm of expression intensity to base 2.

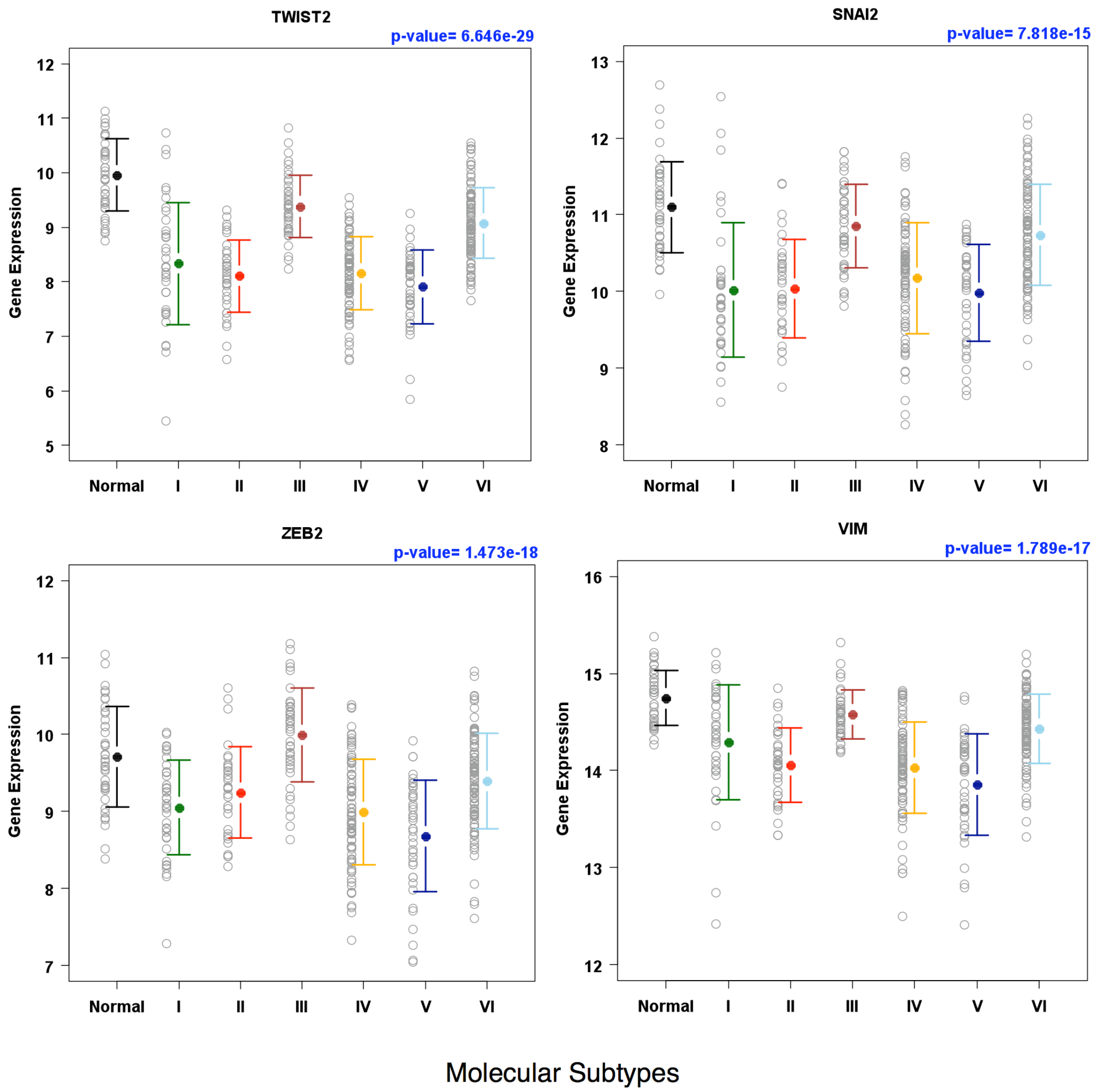

Supplement: Additional file 3 — Supplemental Figures S1-S7. This set of additional files includes the following six supplemental figures. Figure S1 Cut-points to determine positivity of ER, PR and HER2. Figure S2 Correlation studies between immunohistochemistry and gene expression results for ER, PR and HER2 statuses. Figure S3 Functional annotation of gene clusters for breast cancer molecular subtyping. Figure S4 Dendrograms of genes associated with cell cycle/proliferation, stromal reaction, wound response and vascular endothelial normalization for characterizing breast cancer molecular subtypes. Figure S5 Differential expression of the selected genes by breast cancer molecular subtypes in different datasets. Figure S6 Comparison of metastasis-free survival between Subtypes V and VI breast cancer patients classified as Perou-Sørlie luminal A intrinsic type in patients of the present study. Figure S7 Differential expression of genes associated with epithelial-mesenchymal transition among breast cancer molecular subtypes of the present study. [file 1471-2407-11-143-S3.PDF]
